# Clinical characteristics and emergency department-based warning indicators of fulminant myocarditis in children

**DOI:** 10.1371/journal.pone.0358335

**Published:** 2026-09-17

**Authors:** Lingzhu Huang, Jingbo Jiang, Ying Qi, Xiaoxin Deng, Weike Ma, Xiaozhou Chen, Ping Song

**Affiliations:** 1 Department of Emergency Medicine, Shenzhen Children’s Hospital, Shantou University Medical College, Shenzhen, China; 2 Department of Neonatology, Shenzhen Children’s Hospital, Shantou University Medical College, Shenzhen, China; 3 Pediatric Intensive Care Unit, Shenzhen Children’s Hospital, Shantou University Medical College, Shenzhen, China; 4 Department of Pediatric Cardiology, Shenzhen Children’s Hospital, Shantou University Medical College, Shenzhen, China; Mansoura University Faculty of Medicine, EGYPT

## Abstract

**Background:**

Fulminant myocarditis (FM) in children is associated with a high mortality rate, while its early clinical manifestations are often nonspecific. This study aimed to identify early warning indicators available at emergency department (ED) presentations to facilitate early recognition of children at high risk of FM.

**Methods:**

A retrospective analysis was conducted on 140 children with acute myocarditis admitted through the ED between 01/01/2015 and 31/12/2024. Patients were classified into acute non-fulminant myocarditis (ANFM) (n = 86) and FM (n = 54) groups. The least absolute shrinkage and selection operator (LASSO) regression was utilized to optimize variable selection, followed by multivariable logistic regression analysis to identify early warning indicators of FM.

**Results:**

Gastrointestinal symptoms were the most common clinical manifestation in children with FM (88.9%). Compared with the ANFM group, children with FM had lower serum sodium levels and higher lactate and myocardial injury marker levels (all *P* < 0.05). Multivariable logistic regression identified five early warning indicators of FM: gastrointestinal symptoms (OR = 11.468, 95% CI: 2.495–52.713), lactate (OR = 1.887, 95% CI: 1.155–3.082), cardiac troponin I (cTnI) (OR = 1.188, 95% CI: 1.055–1.337), third-degree atrioventricular block (OR = 32.211, 95% CI: 2.943–352.484), and serum sodium (OR = 0.785, 95% CI: 0.652–0.944).

**Conclusions:**

Elevated lactate and cTnI, decreased serum sodium, gastrointestinal symptoms, and third-degree atrioventricular block at ED presentation may help identify children at high risk of FM. Prospective multicenter studies are needed to validate these findings.

## Introduction

Myocarditis is an inflammatory disease of the myocardium with clinical manifestations that vary widely, ranging from mild flu-like symptoms to syncope, seizures, heart failure, cardiogenic shock, and even sudden cardiac death [1]. Pediatric myocarditis may lead to severe long-term sequelae, such as chronic congestive heart failure, dilated cardiomyopathy (DCM), and even death [2–4]. Previous studies have estimated the annual incidence of pediatric myocarditis to range from 0.3 to 2 per 100,000 children [5–8]. According to the 2023 guidelines of the Japanese Circulation Society (JCS), approximately 30%–40% of pediatric myocarditis cases present as fulminant myocarditis (FM), while 40%–65% are classified as acute non-fulminant myocarditis (ANFM) [8]. FM is characterized by an insidious onset, rapid clinical deterioration, and an extremely high mortality risk. Early recognition and prompt initiation of aggressive circulatory support have been shown to improve clinical outcomes [9].

As the emergency department (ED) serves as the initial point of medical contact for most children, the early identification of those at high risk for FM from nonspecific symptoms and signs is critically important. Emergency physicians often need to make rapid clinical judgments based on limited information, including the initial clinical presentation, laboratory findings, and imaging results [10]. This study retrospectively analyzed the early clinical characteristics of children with ANFM and FM, aiming to identify associated indicators of FM based on parameters available at ED presentation, thereby providing evidence to support rapid clinical decision-making, including patient management, levels of care or referral.

## Materials and methods

### Study population

This single-center retrospective study included children aged 28 days to 16 years who were admitted through the ED of Shenzhen Children’s Hospital between 01/01/2015 and 31/12/2024 with a primary discharge diagnosis of ANFM or FM. Patients were either directly admitted from the ED of our institution or transferred from other hospitals to the ED and subsequently hospitalized.

Potential cases were initially identified through the hospital medical record database using International Classification of Diseases, 10th Revision (ICD-10) codes for myocarditis (I40 and I51.4). All candidate cases were independently reviewed by a pediatric emergency physician and a pediatric cardiologist, both blinded to study outcomes. Discrepancies were resolved by consensus after joint discussion. Patients were classified into the ANFM and FM groups according to the final discharge diagnosis. The study protocol was approved by the Ethics Committee of Shenzhen Children’s Hospital (Approval No. 2025049). Data were accessed for research purposes between 27/06/2025 and 30/08/2025. Identifiable information was removed prior to data analysis. Because this study involved retrospective data collection from existing medical records, the institutional review board waived the requirement for informed consent.

### Diagnostic criteria

The diagnosis of ANFM was established by pediatric cardiology specialists, based on the 2013 European Society of Cardiology (ESC) position statement for clinically suspected myocarditis [11], with adaptations for pediatric patients. ANFM required at least one compatible clinical manifestation, which may include chest pain, dyspnea, palpitations, signs of heart failure, unexplained arrhythmia, or age-appropriate objective findings in young children such as feeding difficulties, lethargy or poor responsiveness, syncope or near syncope, tachycardia, and tachypnea. Additionally, at least one diagnostic criterion from a different category must be met, including: (1) abnormalities on electrocardiography or Holter monitoring; (2) elevated biomarkers of myocardial injury (cardiac troponin I); or (3) structural and/or functional abnormalities detected by echocardiography or cardiac magnetic resonance (CMR), or tissue characterization on CMR demonstrating a typical myocarditis pattern, such as myocardial edema and/or late gadolinium enhancement (LGE).

Based on published international and national guidelines and previous studies, FM was defined as acute myocarditis with rapid hemodynamic deterioration within 2 weeks of symptom onset, manifested as cardiogenic shock or life-threatening arrhythmias with hemodynamic instability, and requiring vasoactive agents and/or mechanical circulatory support [9,12,13].

As CMR was not routinely performed before discharge in most patients, and endomyocardial biopsy (EMB) was not available, the study population consisted of clinically suspected myocarditis cases diagnosed based on a comprehensive assessment of clinical presentation, laboratory findings, electrocardiography, echocardiography, and available CMR results.

### Exclusion criteria

Patients were excluded from the study if they presented with congenital heart disease, cardiomyopathy, coronary or valvular disease, hyperthyroidism, inherited metabolic disorders, or other non-myocarditis causes of cardiac dysfunction; if symptom onset occurred more than 30 days prior to presentation; if they were transferred after more than 24 hours of treatment at another institution; or if myocarditis was listed as a secondary diagnosis.

### Data collection

Candidate variables were selected based on clinical relevance, prior literature, and availability at initial medical contact. All variables were derived from data obtained at the first medical encounter. For patients presented directly to our ED, the earliest available clinical and laboratory data were recorded. For transferred patients, data obtained at the referring hospital prior to transfer were prioritized. For examinations not completed during the ED visit (e.g., echocardiography), the earliest available results on the day of admission were included.

Baseline data included age, sex, duration of illness prior to admission, and clinical outcomes. Clinical manifestations were categorized as respiratory, gastrointestinal, cardiovascular, and neurological symptoms, as well as fever and hepatomegaly. Laboratory and imaging variables included white blood cell count (WBC), C-reactive protein (CRP), serum sodium (Na) and potassium (K), lactate (Lac), myocardial injury markers, electrocardiography, chest radiography, and echocardiography.

### Statistical analysis

Continuous variables with a normal distribution were expressed as mean ± standard deviation and compared between groups using the independent-samples *t*-test. Non-normally distributed continuous variables were presented as median (interquartile range [IQR]) and compared using the Mann–Whitney *U* test. Categorical variables were expressed as counts (percentages) and compared using the chi-square test or Fisher’s exact test. Given the low proportion of missing data (<5%), missing values were imputed using the median for continuous variables and the mode for categorical variables.

Univariable logistic regression analysis was performed to identify candidate variables (*P* < 0.10). Echocardiography was not available for all patients at ED presentation, but LVEF was considered clinically important. Therefore, least absolute shrinkage and selection operator (LASSO) regression analyses were conducted both with and without LVEF. Variables were selected using LASSO regression with 10-fold cross-validation based on the λ.1se criterion.

Given the limited number of events, the number of variables included in the final multivariable logistic regression was restricted according to the events-per-variable principle (EPV ≥ 10). Among variables selected by LASSO, indicators were prioritized based on their relative contribution (as reflected by the absolute magnitude of regression coefficients), and the final set of variables was determined within the EPV constraint.

LASSO is a penalized regression method that performs variable selection by shrinking less informative coefficients toward zero, thereby reducing multicollinearity and overfitting when multiple candidate variables are analyzed.

Selected variables were subsequently entered into multivariable logistic regression, and those with *P* < 0.05 were considered significantly associated with FM.

The discriminative ability of the selected indicators was evaluated using receiver operating characteristic (ROC) curves and the area under the curve (AUC). Calibration was assessed using calibration curves and the Hosmer–Lemeshow test. Internal validation was performed using bootstrap resampling (1,000 iterations) to obtain optimism-corrected AUC values.

Univariable and multivariable logistic regression analyses were performed using SPSS (version 27.0), while LASSO regression, ROC analysis, and internal validation were conducted using R (version 4.5.1).

## Results

### Comparison of baseline and clinical characteristics between FM and ANFM

Between 01/01/2015 and 31/12/2024, 178 children with acute myocarditis were admitted through the ED. After excluding children transferred from other hospitals after more than 24 hours of inpatient treatment (n = 14), those with myocarditis as a secondary diagnosis (n = 17), a previous history of myocarditis (n = 4), chronic myocarditis (n = 2), and congenital heart disease (n = 1), 140 children were included in the final analysis. Among these, 54 children were classified as FM and 86 as ANFM. Children with FM were younger than those with ANFM (6 years vs. 10 years, *P* < 0.001). The FM group had a median symptom duration of 2 days before admission, and all five deaths occurred within 72 hours of admission (Table 1).

**Table 1:** Baseline and clinical characteristics of children with fulminant myocarditis and acute non-fulminant myocarditis.

Variable	FM (n = 54)	ANFM (n = 86)	Total (n = 140)	*P* Value
Age, years, median (IQR)	6 (4, 9)	10 (6, 12)		<0.001
Male, n (%)	24 (44.4)	66 (76.7)	90 (64.2)	<0.001
Symptom duration before admission, days, median (IQR)	2 (1, 3)	2 (1, 4)		0.934
Length of stay, days, median (IQR)	15.5 (12.8, 25)	7 (5, 9)		<0.001
ECMO use, n (%)	19 (35.2)	0 (0.0)	19 (13.6)	<0.001
Mortality, n (%)	5 (9.3)	0 (0.0)	5 (3.6)	0.007
Fever, n (%)	35 (64.8)	34 (39.5)	69 (49.3)	0.004
Hepatomegaly, n (%)	21 (38.9)	5 (5.8)	26 (18.6)	<0.001
Respiratory, n (%)	18 (33.3)	31 (36.0)	49 (35.0)	0.743
Cough	8 (14.8)	25 (29.1)	33 (23.6)	0.053
Dyspnea and/or tachypnea	12 (22.2)	3 (3.5)	15 (10.7)	<0.001
Gastrointestinal, n (%)	48 (88.9)	35 (40.7)	83 (59.3)	<0.001
Nausea and/or vomiting	43 (79.6)	27 (31.4)	70 (50.0)	<0.001
Abdominal pain	26 (48.1)	9 (10.5)	35 (25.0)	<0.001
Diarrhea	2 (3.7)	7 (8.1)	9 (6.4)	0.298
Cardiovascular, n (%)	44 (81.5)	73 (84.9)	117 (83.6)	0.597
Chest pain	5 (9.3)	56 (65.1)	61 (43.6)	<0.001
Chest tightness	7 (13.0)	28 (32.6)	35 (25.0)	0.009
Pallor	23 (42.6)	6 (7.0)	29 (20.7)	<0.001
Fatigue	17 (31.5)	12 (14.0)	29 (20.7)	0.013
Neurological, n (%)	31 (57.4)	26 (30.2)	57 (40.7)	0.001
Dizziness	12 (22.2)	15 (17.4)	27 (19.3)	0.485
Headache	7 (13.0)	7 (8.1)	14 (10.0)	0.354
Seizure	13 (24.5)	3 (3.5)	16 (11.5)	<0.001
Syncope	5 (9.3)	2 (2.3)	7 (5.0)	0.067

Abbreviations: FM, fulminant myocarditis; ANFM, acute non-fulminant myocarditis; ECMO, extracorporeal membrane oxygenation; IQR, interquartile range.

**Fig 1:**
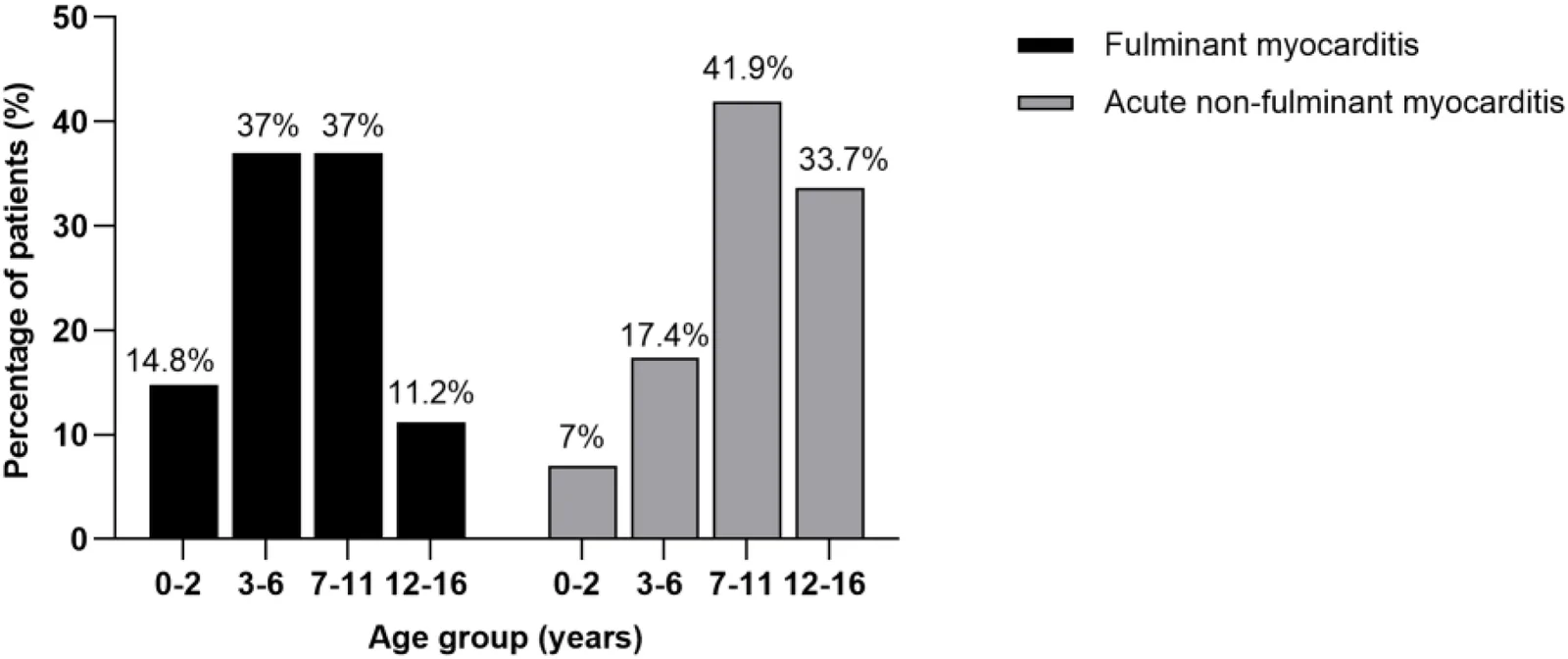
Age distribution of children with fulminant myocarditis and acute non-fulminant myocarditis.

FM occurred most frequently in preschool-aged (3–7 years) and school-aged (7–12 years) children (Fig 1). The proportion of males was higher in the ANFM group than in the FM group, whereas the sex distribution in the FM group was relatively balanced. Gastrointestinal symptoms were more common in the FM group than in the ANFM group (88.9% vs. 40.7%, *P <* 0.001). Among children with FM, nausea and/or vomiting were the most common gastrointestinal manifestations, occurring in 79.6% (43/54) of cases, whereas chest pain was reported in only 9.3% (5/54). Compared with the ANFM group, children with FM had a longer hospital stay (15.5 days vs. 7 days, *P* < 0.001), a higher rate of ECMO use (35.2% vs. 0.0%, *P* < 0.001), and higher mortality (9.3% vs. 0.0%, *P* = 0.007) (Table 1). Age-group distributions of clinical symptoms in children with FM and ANFM are shown in S1 Fig.

### Comparison of laboratory and imaging examinations between FM and ANFM

Compared with the ANFM group, children with FM had significantly lower Na levels and higher Lac and myocardial injury marker levels, including cardiac troponin I (cTnI), lactate dehydrogenase (LDH), creatine kinase-MB (CK-MB), aspartate aminotransferase (AST), and creatine kinase (CK) (all *P <* 0.05) (Table 2).

**Table 2:** Laboratory, electrocardiographic, imaging, and echocardiographic findings in children with fulminant myocarditis and acute non-fulminant myocarditis.

**Variable**	**FM (n=54)**	**ANFM (n=86)**	***P* Value**
WBC, ×10^9^/L, median (IQR)	10.7 (7.7, 15.2)	9.2 (7.0, 11.7)	0.042
CRP, mg/L, median (IQR)	3.9 (1.8, 12.8)	8.7 (1.2, 20.6)	0.158
Na, mmol/L, mean (SD)	134.1± 4.9	137.8 ± 2.0	<0.001
K, mmol/L, median (IQR)	4 (3.6, 4.7)	3.8 (3.6, 4)	0.007
Lac, mmol/L, median (IQR)	3.7 (2.2, 7.6)	1.5 (1.1, 1.6)	<0.001
cTnI, ng/mL, median (IQR)	4.5 (1.5, 24.4)	1.3 (0.1, 4.4)	<0.001
LDH, U/L, median (IQR)	572.5 (408.5, 1082)	294 (243.7, 402)	<0.001
CK-MB, ng/mL, median (IQR)	56.8 (24.4, 97.2)	13.65 (4.1, 41.8)	<0.001
AST, U/L, median (IQR)	139 (88, 457.5)	46 (28, 65.2)	<0.001
CK, U/L, median (IQR)	741.8 (378.3, 1648.7)	277.5 (136.9, 578.2)	<0.001
Cardiomegaly on chest radiography, n (%)	22 (40.7)	10 (11.6)	<0.001
CMR, n (%)	13 (24.1)	33 (38.4)	0.080
Abnormal ECG findings, n (%)	50 (92.6)	62 (72.1)	0.003
T-wave abnormalities	11 (20.4)	15 (17.4)	0.664
ST-segment abnormalities	20 (37.0)	35 (40.7)	0.666
Bundle branch block	13 (24.1)	13 (15.1)	0.185
AVB	20 (37.0)	5 (5.8)	<0.001
First-degree AVB	4 (7.4)	2(2.3)	0.205
Second-degree AVB	1 (1.9)	1 (1.2)	0.624
Third-degree AVB	15 (27.8)	2 (2.3)	<0.001
Ventricular tachycardia	5 (9.3)	0 (0.0)	0.008
Abnormal Q wave	8 (14.8)	6 (7.0)	0.132
Echocardiographic findings, n (%)			
Ventricular wall thickening	15 (27.8)	12 (14.0)	0.044
Chamber dilation	12 (22.2)	5 (5.8)	0.004
Wall motion abnormalities	33 (61.1)	12 (14.0)	<0.001
Pericardial effusion	17 (31.5)	8 (9.3)	0.001
LVEF, %, median (IQR)	47.5 (41, 55.2)	64.1 (60, 69.8)	<0.001

Abbreviations: FM, fulminant myocarditis; ANFM, acute non-fulminant myocarditis; WBC, white blood cell count; CRP, C-reactive protein; Na, serum sodium; K, serum potassium; Lac, lactate; cTnI, cardiac troponin I; LDH, lactate dehydrogenase; CK-MB, creatine kinase-MB; AST, aspartate aminotransferase; CK, creatine kinase; ECG, electrocardiography; AVB, atrioventricular block; LVEF, left ventricular ejection fraction; CMR, cardiac magnetic resonance; SD, standard deviation; IQR, interquartile range.

Electrocardiographic abnormalities were observed in 92.6% of children with FM, with ST-segment changes (37.0%) and atrioventricular block (AVB) (37.0%) being the most common findings. Compared with the ANFM group, the FM group had a higher incidence of abnormal echocardiographic findings, including ventricular wall thickening, chamber dilation, wall motion abnormalities, and pericardial effusion. Furthermore, LVEF was significantly lower in the FM group (47.5% vs. 64.1%, *P <* 0.05). Cardiomegaly on chest radiography was also more frequent in the FM group (*P <* 0.05) (Table 2). CMR was performed in 46 patients, 29 of whom showed findings consistent with myocarditis. CMR was performed at a median of 7 (5–9) days after ED presentation, with a range of 2–22 days.

### Warning indicators for early identification of FM in the ED

In the analysis excluding LVEF, variables were entered into LASSO regression with 10-fold cross-validation (*λ.*1se = 0.031).

**Fig 2:**
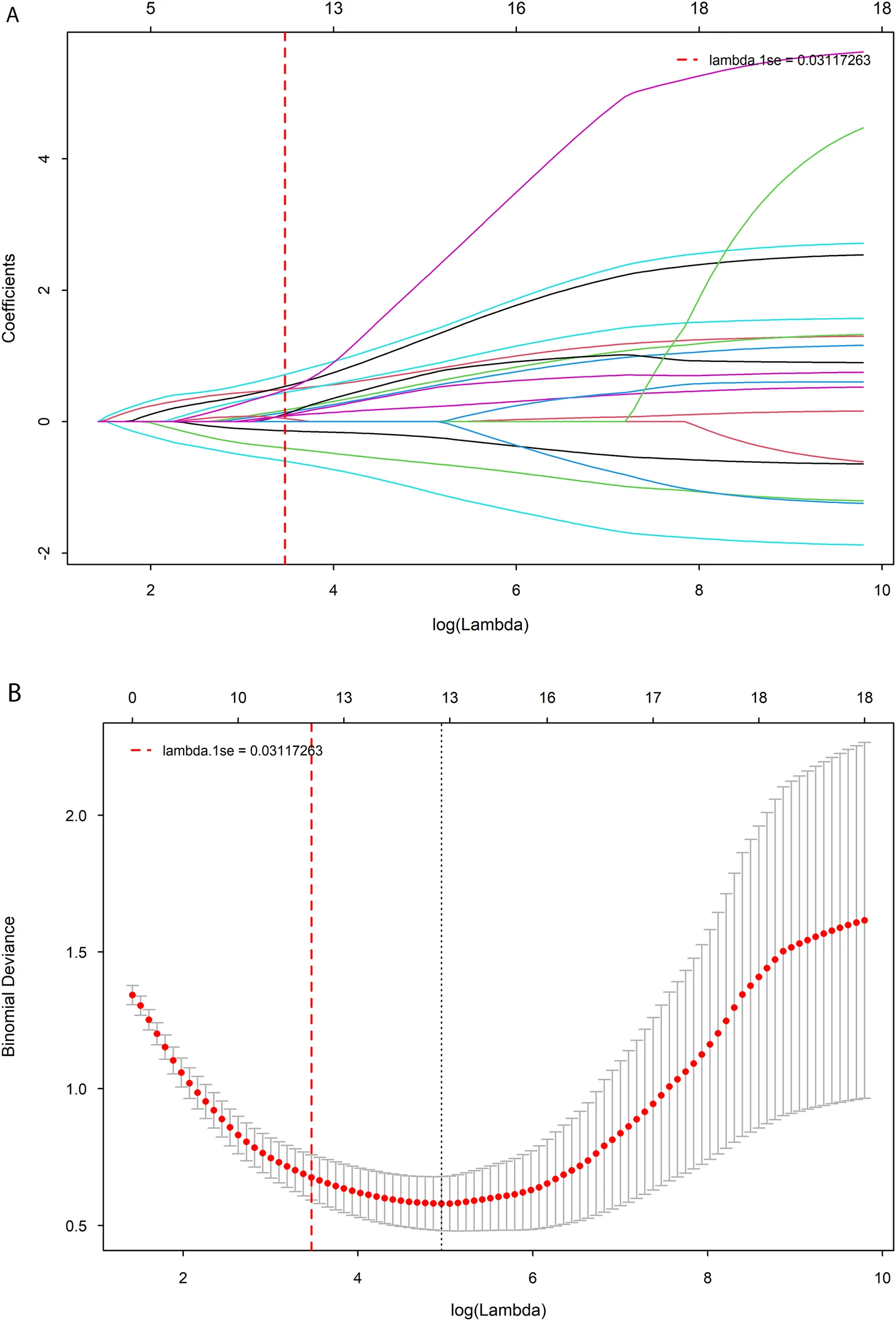
Least absolute shrinkage and selection operator variable selection for indicators of fulminant myocarditis. (A) Coefficient regularization paths of candidate variables. As the penalty parameter λ increases, coefficients shrink toward zero. The red dashed line indicates the value of *λ.*1se = 0.031, at which 14 variables with non-zero coefficients were retained. (B) Ten-fold cross-validation curve for selection of the optimal penalty parameter *λ.* The red dashed line indicates *λ.*1se = 0.031.

A total of 14 variables with non-zero coefficients were identified, including Lac, Na, third-degree AVB, cTnI, gastrointestinal symptoms, age, hepatomegaly, neurological symptoms, fever, CK-MB, male, glucose, cardiomegaly, and LDH (Fig 2A and 2B).

Based on the EPV ≥ 10 principle (EPV = 10.8) and variable contribution, the five variables with the largest absolute coefficients were included in the multivariable logistic regression analysis. Ultimately, five warning indicators of FM were identified (Table 3): gastrointestinal symptoms (OR = 11.468, 95% CI: 2.495–52.713, *P* = 0.002), Lac (OR = 1.887, 95% CI: 1.155–3.082, *P* = 0.011), cTnI (OR = 1.188, 95% CI: 1.055–1.337, *P* = 0.004), third-degree AVB (OR = 32.211, 95% CI: 2.943–352.484, *P* = 0.004), and Na (OR = 0.785, 95% CI: 0.652–0.944, *P* = 0.010) (Table 3).

**Table 3:** Multivariable logistic regression analysis of factors associated with fulminant myocarditis at emergency department presentation.

Variable	Coefficient	OR	95% CI	*P* value
Gastrointestinal symptoms	2.440	11.468	2.495-52.713	0.002
Lac	0.635	1.887	1.155-3.082	0.011
cTnI	0.172	1.188	1.055-1.337	0.004
Third-degree AVB	3.472	32.211	2.943-352.484	0.004
Na	−0.242	0.785	0.652-0.944	0.010

Abbreviations: Lac, lactate; cTnI, cardiac troponin I; AVB, atrioventricular block; Na, serum sodium; OR, odds ratio; CI, confidence interval.

**Fig 3:**
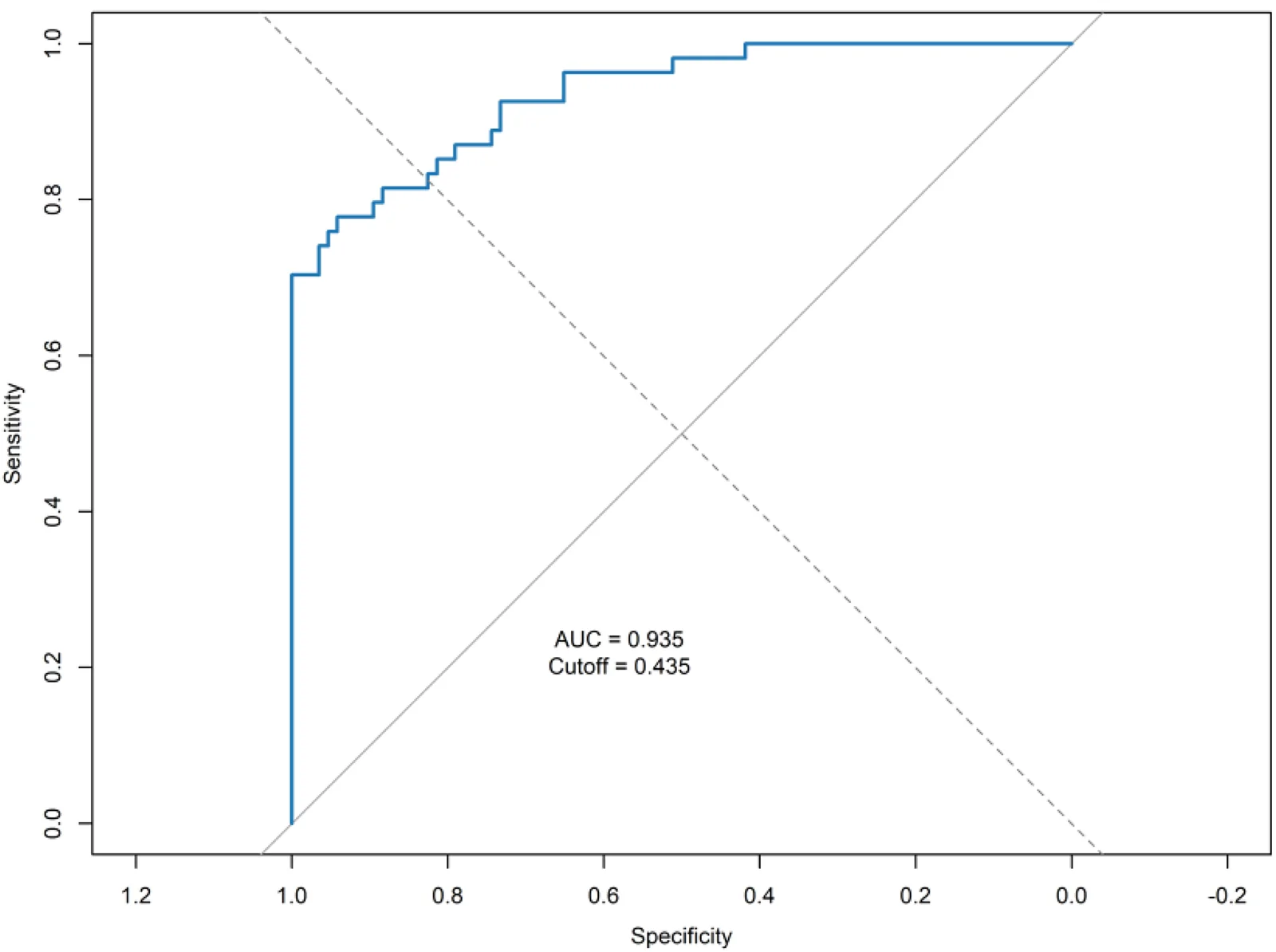
Receiver operating characteristic curve of the selected early warning indicator combination for fulminant myocarditis. Abbreviation: ROC, receiver operating characteristic curve.

**Fig 4:**
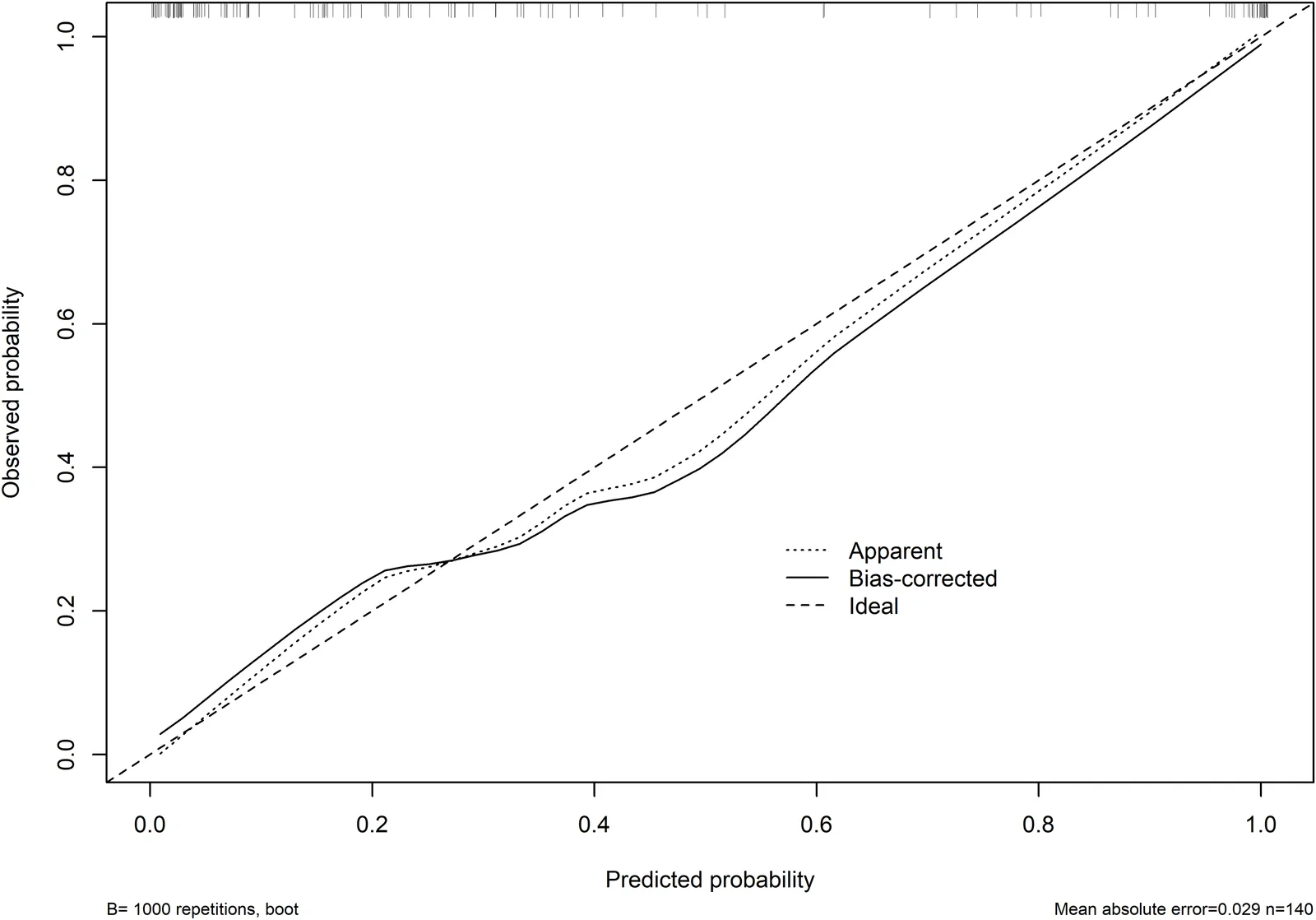
Calibration curve of the combined early warning indicators for fulminant myocarditis.

The combination of warning indicators showed good discriminatory ability, with an AUC of 0.935 (Fig 3). The optimal cutoff value was 0.435, with a sensitivity of 0.777, specificity of 0.941, positive predictive value of 0.893, and negative predictive value of 0.871. After bootstrap internal validation (B = 1,000), the optimism-corrected AUC was 0.918. Calibration curves showed good agreement between predicted and observed probabilities (Fig 4). The Hosmer–Lemeshow test indicated good fit (χ² = 2.469, *P* = 0.963).

In a supplementary analysis including LVEF when available (S1 Table), the AUC increased to 0.973, indicating improved discrimination (S2 Fig).

## Discussion

Our institution is a tertiary pediatric referral center with an annual ED volume exceeding 200,000 visits, receiving critically ill children transferred from both the local city and surrounding regions. Using a combination of LASSO regression and multivariable logistic regression, this study explored warning indicators of FM at ED presentations. We identified elevated Lac and cTnI, decreased Na, gastrointestinal symptoms, and third-degree AVB as warning indicators associated with FM. Importantly, all the variables can be readily obtained during initial ED evaluation. These findings may assist pediatric emergency physicians in swiftly identifying children at high risk of progressing to FM and facilitate timely therapeutic interventions.

Two nationwide epidemiological surveys conducted in Japan at a 10-year interval reported an overall survival rate of 75.6% among children with myocarditis, whereas the survival rate of FM remained substantially lower, ranging from 48.6% to 51.6% [1,14]. Some children with FM present to the ED with hemodynamic instability or overt shock, forcing emergency physicians to make urgent clinical decisions in the absence of sufficient diagnostic evidence. Furthermore, ANFM may rapidly progress to hemodynamic deterioration and refractory heart failure. Previous studies have demonstrated that early identification of FM, prompt initiation of mechanical circulatory support, and aggressive maintenance of organ function are associated with favorable outcomes in pediatric myocarditis [9,14].

When cardiac function deteriorates rapidly, resulting in a marked reduction in cardiac output, inadequate peripheral perfusion and increased anaerobic metabolism lead to excessive Lac production. Therefore, Lac may help assess disease severity in children with myocarditis. In our study, Lac levels were significantly higher in the FM group. Multivariable logistic regression further demonstrated that for each 1 mmol/L increase in Lac, the odds of FM increased by approximately 1.9-fold, supporting Lac as a warning indicator associated with FM during ED evaluation. A nationwide cohort study in Japan reported that Lac ≥ 2 mmol/L at admission was associated with a higher likelihood of receiving venoarterial extracorporeal membrane oxygenation (VA-ECMO) [15]. Additionally, a multicenter retrospective study by Nichol *et al*. demonstrated that hyperlactatemia was independently associated with increased mortality among critically ill patients [16]. These findings suggest that elevated Lac may reflect greater disease severity and poorer clinical outcomes.

Our study also identified gastrointestinal symptoms as important warning indicators of FM. This may be attributed to systemic hypoperfusion and metabolic acidosis caused by FM, leading to gastrointestinal ischemia. Gastrointestinal symptoms may therefore reflect severe disease progression in pediatric myocarditis. Butts *et al*. reported that gastrointestinal symptoms at hospital admission in children with myocarditis were associated with an increased risk of death or heart transplantation [17]. Similarly, Fatma *et al*. demonstrated that vomiting was more common among children with acute myocarditis who subsequently died [18]. Previous studies have also indicated that a considerable proportion of myocarditis patients presenting with abdominal pain developed circulatory failure within the first week. These findings suggest that pediatric emergency physicians should remain vigilant when gastrointestinal symptoms are accompanied by signs of hypoperfusion, as this combination may indicate an early manifestation of FM.

cTnI is an important biomarker of myocardial injury, with higher sensitivity and specificity than traditional markers such as CK-MB and LDH, but normal cTnI levels do not exclude myocarditis [8]. Elevated cTnI levels have been associated with the need for ECMO and increased mortality [19]. In our study, initial cTnI levels were significantly higher in children with FM, consistent with findings from a nationwide Japanese study [2], suggesting its potential value for early risk stratification at ED presentation. However, previous studies have shown that cTnI may also be markedly elevated in children with only mild ventricular dysfunction and do not always correlate linearly with disease severity [17–19]. Therefore, cTnI should be interpreted in combination with other indicators. Although CK-MB and LDH were initially retained in LASSO selection, they were excluded from the final multivariable analysis due to their relatively lower contribution under the prespecified EPV constraint.

Our study identified decreased Na as an early warning indicator of FM. For each 1 mmol/L decrease in Na, the odds of FM increased by approximately 27%. The underlying mechanisms likely involve pro-inflammatory cytokines stimulating the release of arginine vasopressin (AVP), along with tissue hypoperfusion triggering non-osmotic AVP secretion [20,21]. A retrospective observational study involving 1,932 ED patients demonstrated a significantly higher mortality among those with hyponatremia [22]. A large cohort study found that when Na levels fell below 138 mmol/L, each 1 mmol/L decrease was linked to an 8% increase in the risk of in-hospital mortality [20]. These findings suggest that Na levels should be closely monitored in children with suspected acute myocarditis, as a decrease in Na may indicate progression toward FM.

AVB is a prevalent and serious arrhythmia in children with FM [23]. It reflects direct inflammatory injury to the cardiac conduction system, potentially resulting from myocardial cell swelling or injury. AVB is also frequently associated with left ventricular dysfunction and heart failure, indicating advanced myocardial involvement [24]. In our study, children with third-degree AVB in the ED had approximately 32-fold higher odds of FM. Although the absolute incidence of third-degree AVB was not the highest (being lower than the 88.9% for gastrointestinal symptoms), its association with FM (as reflected by the OR value) was indeed the strongest. Previous studies have also reported that third-degree AVB is an important indicator of FM, allowing timely cardiac evaluation and therapeutic intervention [25]. Given that electrocardiography is a readily available bedside examination in the ED, and that third-degree AVB showed a strong association with FM, it may serve as a key early warning indicator for the initial recognition of FM.

FM may present with cardiogenic shock, and hypotension often draws immediate clinical attention. However, our study focused on early warning indicators preceding overt circulatory failure rather than on constructing a predictive model. Consequently, hypotension was not included in the analysis, although children with cardiogenic shock or low cardiac output were not excluded.

The higher proportion of males in the ANFM group may be related to sex hormone–mediated differences in immune responses, with androgens potentially promoting stronger pro-inflammatory responses and estrogens favoring anti-inflammatory and reparative processes [26,27]. Children with FM were also younger, which may reflect differences in clinical presentation. Younger children often rely on parental observation for non-specific symptoms, whereas older children can more clearly describe cardiovascular symptoms, enabling timely consultation. Although sex and age were selected by LASSO, they were not retained in the multivariable analysis. This may be due to their relatively lower contribution under the EPV constraint, the absence of significant sex differences within the FM group, and potential overlap between age and clinical presentation.

Early identification of FM may help facilitate timely targeted treatment, improve the likelihood of successful rescue, and reduce the risk of mortality [13]. Previous studies have reported factors associated with a fulminant course in acute myocarditis. Kato *et al*. identified elevated CRP, CK, reduced LVEF, and intraventricular conduction disturbances as independent risk factors for fulminant progression [28]. Inaba *et al*. reported that low systolic blood pressure and prolonged QRS duration at admission were important predictors of a fulminant course [29]. In contrast, our study focused on the early identification of FM at ED presentation. The indicator combination identified in this study demonstrated good discriminative ability and stability after internal validation, and all included variables are readily obtainable in the ED setting. Moreover, most variables are derived from routine clinical assessment and laboratory testing, which may be particularly advantageous in primary or resource-limited settings where echocardiography or advanced imaging modalities may not be readily accessible. By facilitating early identification of children at high risk for FM, our findings may assist emergency physicians in making more timely decisions regarding closer monitoring, early escalation of care, or the arrangement of early referral to tertiary centers with advanced circulatory support when appropriate.

Echocardiography is a first-line tool for assessing cardiac structure and function in children with suspected myocarditis. However, due to the retrospective study design and limited availability, echocardiographic data were often not available at ED presentation. Therefore, this study focused on exploring early warning indicators that do not rely on echocardiography. Nevertheless, echocardiography findings remain clinically important. In our cohort, LVEF was significantly lower in the FM group, with a median value below 50%, suggesting that many children with FM had already developed substantial cardiac dysfunction at presentation. Previous studies have shown that reduced LVEF is associated with the need for intensive care [18] and predicts a fulminant disease course [29]. In the supplementary analysis, inclusion of LVEF improved the discriminative ability of the warning indicators for early FM identification. However, even without LVEF, the combination of clinical and laboratory indicators still showed acceptable discrimination, supporting its practical value in the ED setting. In practice, early integration of laboratory findings and timely use of echocardiography, when available, may further improve early recognition of FM.

### Limitations

This study has several limitations. First, as a single-center retrospective study conducted at a tertiary referral center, the findings may be subject to selection bias and limited generalizability, particularly given the relatively high proportion of severe myocarditis cases. External validation in multicenter cohorts is therefore needed. Second, the retrospective design limited data collection to available clinical records, and detailed information regarding in-hospital management, dynamic disease progression, and long-term follow-up was not consistently available. Third, FM and ANFM were diagnosed primarily based on clinical grounds rather than systematic CMR or EMB confirmation, which may compromise diagnostic accuracy and introduce classification bias; variability in the timing of CMR can have a similar effect. Because FM classification relied mainly on rapidly progressive hemodynamic deterioration and the need for advanced life support, some degree of diagnostic misclassification between FM and severe ANFM cannot be excluded. Fourth, although the final analysis was restricted according to the prespecified EPV principle, the number of FM cases remained limited and no external validation was performed. Finally, some identified indicators, such as Lac and third-degree AVB, may partly reflect hemodynamic compromise, leading to potential incorporation bias that should be interpreted cautiously. Future prospective multicenter studies are needed to validate the robustness and generalizability of these findings across diverse populations, age groups, and healthcare settings.

## Conclusions

Elevated Lac and cTnI levels, decreased Na levels, gastrointestinal symptoms, and third-degree AVB were associated with FM at ED presentation. These readily available indicators may facilitate early recognition of children at high risk of FM. Prospective multicenter studies are needed to validate these findings.

## Supporting information

S1 Fig Age-group distribution of clinical symptoms in children with fulminant myocarditis and acute non-fulminant myocarditis. The x-axis shows symptom categories, and the y-axis shows the percentage of patients in each age group with each symptom. Colors indicate different age groups. Percentages are calculated within each age group. Patients may have presented with multiple symptoms; therefore, percentages do not sum to 100%. One asymptomatic case was included (n = 1). Abbreviations: FM, fulminant myocarditis; ANFM, acute non-fulminant myocarditis.
(DOCX)

S1 Table Multivariable logistic regression analysis including left ventricular ejection fraction. (DOCX)

S2 Fig Receiver operating characteristic curve of the supplementary analysis including left ventricular ejection fraction. Abbreviation: ROC, receiver operating characteristic curve.
(DOCX)

S1 Data Anonymized minimal dataset supporting the findings of this study. (XLSX)
