# Supplementary material for: Clinical characteristics and emergency department-based warning indicators of fulminant myocarditis in children
Source: PLoS One. 2026 Sep 17;21(9):e0358335. doi: 10.1371/journal.pone.0358335 (PMC13585240; doi:10.1371/journal.pone.0358335)
Supplement: Supplementary file 1 [file pone.0358335.s001.docx]

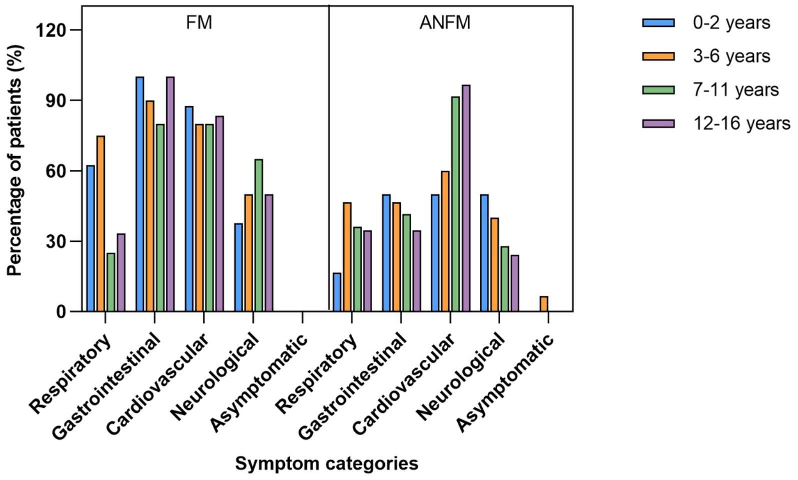
FM
ANFM
0-2 years
3-6 years
7-11 years
12-16 years
Percentage of patients (%)
120
90
60
30
0
Respiratory
Gastrointestinal
Cardiovascular
Neurological
Asymptomatic
Respiratory
Gastrointestinal
Cardiovascular
Neurological
Asymptomatic
Symptom categories
