# Supplementary material for: Clinical characteristics and emergency department-based warning indicators of fulminant myocarditis in children
Source: PLoS One. 2026 Sep 17;21(9):e0358335. doi: 10.1371/journal.pone.0358335 (PMC13585240; doi:10.1371/journal.pone.0358335)
Supplement: Supplementary file 2 [file pone.0358335.s003.docx]

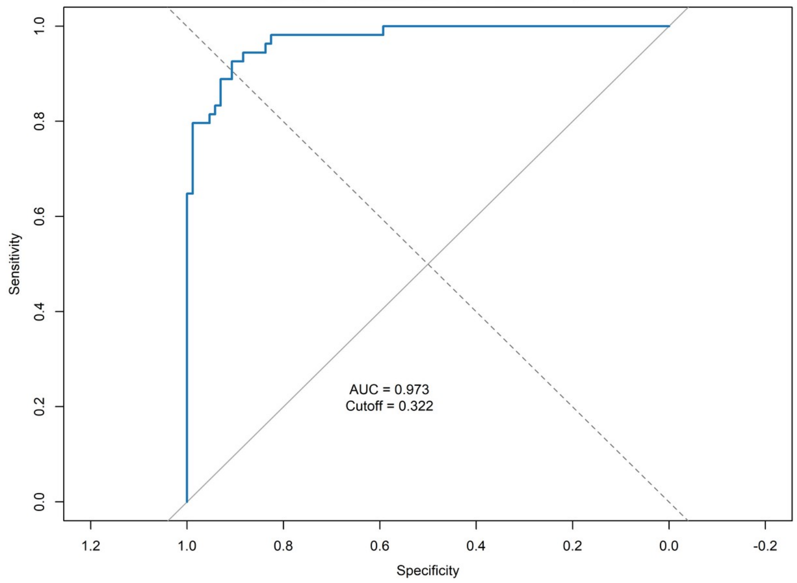

1.0
0.8
0.6
0.4
0.2
0.0
Sensitivity
AUC = 0.973
Cutoff = 0.322
1.2
1.0
0.8
0.6
0.4
0.2
0.0
-0.2
Specificity
