# Supplementary material for: Clinical characteristics and emergency department-based warning indicators of fulminant myocarditis in children
Source: PLoS One. 2026 Sep 17;21(9):e0358335. doi: 10.1371/journal.pone.0358335 (PMC13585240; doi:10.1371/journal.pone.0358335)
Supplement: Supplementary file 2 [file pone.0358335.s002.docx]

**S1 Table. Multivariable logistic regression analysis including left ventricular ejection fraction**

| **Variable** | **Coefficient** | **OR** | **95% CI** | ***P* value** |
| --- | --- | --- | --- | --- |
| Gastrointestinal symptoms | 2.176 | 8.813 | 1.701-45.646 | 0.010 |
| LVEF | -0.188 | 0.828 | 0.765-0.897 | <0.001 |
| Age | -0.034 | 0.966 | 0.946-0.987 | 0.002 |
| Third-degree AVB | 3.829 | 46.004 | 4.517-468.524 | 0.001 |
| Na | -0.229 | 0.795 | 0.654-0.968 | 0.022 |
| Abbreviation: LVEF, left ventricular ejection fraction; AVB, atrioventricular block; Na, serum sodium; OR, odds ratio; CI, confidence interval. | | | | |
