# Supplementary material for: Clinical characteristics and emergency department-based warning indicators of fulminant myocarditis in children
Source: PLoS One. 2026 Sep 17;21(9):e0358335. doi: 10.1371/journal.pone.0358335 (PMC13585240; doi:10.1371/journal.pone.0358335)
Supplement: Supplementary file 4 [file pone.0358335.s006.docx]

Dear Editor and Reviewers,

We sincerely thank the editor and all reviewers for your professional and insightful comments. Your feedback has greatly helped us improve the quality of our manuscript. Below, we provide a detailed point-by-point response and summary of the revisions made in accordance with your comments. The reviewers’ comments are presented in bold, and our responses are provided in regular text. In the revised manuscript, all changes and additions are highlighted in blue.

# Reviewer #1:

1. **Well written manuscript with fairly large number of cases at a single pediatric ED.**

Response: We sincerely thank you for the positive assessment of our manuscript and for acknowledging the value of our relatively large pediatric ED cohort from a single center.

**2. Description of standard statistical analyses clear, but the LASSO method is not commonly seen in pediatric manuscripts, and I would think needs a fuller explanation for most readers.**

Response: Thank you for this helpful suggestion. We agree that LASSO regression may not be familiar to some readers of pediatric manuscripts. Accordingly, we have added a clearer explanation in the Methods section（lines 171-173）.

Revised text: Lines 171-173: LASSO is a penalized regression method that performs variable selection by shrinking less informative coefficients toward zero, thereby reducing multicollinearity and overfitting when multiple candidate variables are analyzed.

**3. A strength of the manuscript is the emphasis on how myocarditis (acute and non-acute) initially presents to an emergency room. But exclusion of cases that have obvious low output/shock symptoms skews the actual proportion of cases of myocarditis that would fall in the "fulminant category".**

Response: Thank you for this important comment. We would like to clarify that patients presenting with shock or low cardiac output were not excluded from this study. All eligible cases were consecutively enrolled. Hypotension was not included in the analysis because our aim was not to construct a clinical prediction model for FM, but rather to explore earlier warning indicators associated with FM before overt circulatory failure becomes clinically apparent. We have revised the manuscript to make this point clearer and to emphasize that the study focuses on early risk recognition, rather than on building a prediction model for FM in the Discussion section（lines 354-358).

Revised text: Lines 354-358: FM may present with cardiogenic shock, and hypotension often draws immediate clinical attention. However, our study focused on early warning indicators preceding overt circulatory failure rather than on constructing a predictive model. Consequently, hypotension was not included in the analysis, although children with cardiogenic shock or low cardiac output were not excluded.

4. **It is understandable that the number of patients with a CMR is limited in this retrospective review, but the fact that only 29 of 46 patients had a positive study confirming the diagnosis merits some discussion. Since a CMR is currently the most reliable diagnostic study (other than the relatively infrequently used biopsy), the accuracy of the diagnosis could be questioned. This concern would be mitigated by giving information on the timing of the CMR compared to ED presentation.**

Response: Thank you for your important comment. We agree that the limited use of CMR and the absence of EMB may raise concerns regarding diagnostic accuracy. In our center, most patients did not undergo CMR prior to discharge, and EMB has not been routinely performed. Therefore, the study population consisted of clinically suspected myocarditis cases diagnosed based on a comprehensive assessment of clinical presentation, laboratory findings, electrocardiography, echocardiography, and available CMR results. We acknowledge that this approach may introduce potential misclassification bias. However, according to the 2021 AHA scientific statement on pediatric myocarditis, a negative CMR does not exclude the diagnosis, and clinically suspected myocarditis may still be diagnosed based on clinical and laboratory evidence. We also agree that the timing of CMR relative to ED presentation may influence its diagnostic yield. CMR is known to be time-sensitive. The 2024 American College of Cardiology (ACC) expert consensus on myocarditis indicates that the detection rate is highest within 1–2 weeks after symptom onset, when myocardial edema and inflammation are most prominent, whereas examinations performed too early or too late may reduce sensitivity. In addition, previous studies have shown that inflammatory changes may still be detected by EMB in patients with negative CMR findings. In the revised manuscript, we have added information on the timing of CMR in the Methods section (lines 224-225). We have also expanded the Discussion section to address this limitation (lines 409-413).

Revised text in Methods: Lines 224-225: CMR was performed at a median of 7 (5-9) days after ED presentation, with a range of 2 to 22 days.

Revised text in Limitations: Lines 409-413: Third, FM and ANFM were diagnosed primarily based on clinical grounds rather than systematic CMR or EMB confirmation, which may compromise diagnostic accuracy and introduce classification bias; variability in the timing of CMR can have a similar effect.

5. **Highlighting the clinical and lab findings that help identify fulminant myocarditis is valuable. However, in the discussion of troponin (page 17, lines 324-341), it important to point out that very high levels can sometimes be found in non-fulminant myocarditis (emphasized in the Scientific Statement by Law, et al - reference 17).**

Response: Thank you for your important comment. We agree that markedly elevated cTnI levels may also be observed in patients with ANFM, and that the degree of troponin elevation does not necessarily correlate linearly with disease severity. Therefore, cTnI should not be interpreted as a standalone marker for risk stratification, but rather in conjunction with clinical presentation and other supportive findings. In the Discussion section, we have clarified that elevated troponin should be interpreted cautiously in the clinical context (lines: 324-327).

Revised text in Discussion: Lines 324-327: However, previous studies have shown that cTnI may also be markedly elevated in children with only mild ventricular dysfunction and do not always correlate linearly with disease severity (17-19). Therefor, cTnI should be interpreted in combination with other indicators.

6. **Presenting symptoms considered should include syncope or near syncope (page 6, lines128-131).**

Response: Thank you for this suggestion. We have added “syncope or near syncope” to the list of presenting symptoms in the Methods section (line 112).

7. **The biomarker of BNP or pro-BNP may be useful at presentation, but it was not mentioned.**

Response: Thank you for your suggestion. BNP or NT-proBNP may indeed be clinically useful at presentation in children with acute myocarditis, as elevated levels are often associated with cardiac dysfunction, acute heart failure, and the need for resuscitation or mechanical circulatory support. However, because this was a retrospective study based on data obtained at the time of ED presentation, BNP/NT-proBNP testing was not routinely performed in a large proportion of patients, resulting in substantial missing data. Therefore, these biomarkers could not be reliably included in the present analysis. The Limitations section has addressed this limitation (line 407-409).
Revised text in Limitations: Lines 407-409: Second, the retrospective design limited data collection to available clinical records, and detailed information regarding in-hospital management, dynamic disease progression, and long-term follow-up was not consistently available.

8. **The importance of AV block is a key finding; but it needs to be broken down into the degrees of block.**

Response: Thank you very much for this valuable suggestion. In response to your comment, we further categorized AVB by degree (first-, second-, and third-degree AVB) and added the comparison of different AVB grades between the FM and non-FM groups in the revised manuscript (Table 2). The results showed that third-degree AVB was substantially more frequent in the FM group than in the ANFM group. Based on this finding, third-degree AVB was entered as a variable in the LASSO regression and multivariable logistic regression analyses, where it remained significantly associated with FM (Results: lines 236-239, 246-252). We have also revised the Discussion to emphasize its potential value as an early warning sign of FM (lines 345-353).

Revised text in Results:

Table 2：

| **Variable** | **FM (n=54)** | **ANFM (n=86)** | ***P* Value** |
| --- | --- | --- | --- |
| First-degree AVB | 4 (7.4) | 2(2.3) | 0.205 |
| Second-degree AVB | 1 (1.9) | 1 (1.2) | 0.624 |
| Third-degree AVB | 15 (27.8) | 2 (2.3) | <0.001 |

Lines 236-239: A total of 14 variables with non-zero coefficients were identified, including Lac, Na, third-degree AVB, cTnI, gastrointestinal symptoms, age, hepatomegaly, neurological symptoms, fever, CK-MB, male, glucose, cardiomegaly, and LDH (Fig 2A and 2B).

### Lines 246-252: Based on the EPV ≥ 10 principle (EPV = 10.8) and variable contribution, the five variables with the largest absolute coefficients were included in the multivariable logistic regression analysis. Ultimately, five warning indicators of FM were identified (Table 3): gastrointestinal symptoms (OR = 11.468, 95% CI: 2.495–52.713, *P* = 0.002), Lac (OR = 1.887, 95% CI: 1.155–3.082, *P* = 0.011), cTnI (OR = 1.188, 95% CI: 1.055–1.337, *P* = 0.004), third-degree AVB (OR = 32.211, 95% CI: 2.943–352.484, *P* = 0.004), and Na (OR = 0.785, 95% CI: 0.652–0.944, *P* = 0.010)（Table 3).

Revised text in Discussion: Lines 345-353: In our study, children with third-degree AVB in the ED had approximately 32-fold higher odds of FM. Although the absolute incidence of third-degree AVB was not the highest (being lower than the 88.9% for gastrointestinal symptoms), its association with FM (as reflected by the OR value) was indeed the strongest. Previous studies have also reported that third-degree AVB is an important indicator of FM, allowing timely cardiac evaluation and therapeutic intervention (25). Given that electrocardiography is a readily available bedside examination in the ED, and that third-degree AVB showed a strong association with FM, it may serve as a key early warning indicator for the initial recognition of FM.

9. **There is no mention of the follow up of the patients in this series, both short and longer term follow if available would-be important information to put the diagnosis of fulminant and non-fulminant in perspective.**
   Response: Thank you for this valuable suggestion. We attempted to collect follow-up information during data collection. However, due to the retrospective nature of the study and the fact that many patients were referred from surrounding regions, complete follow-up after discharge could not be consistently obtained. As a result, both short-term and long-term follow-up data were largely incomplete and were therefore not included in the present analysis. We acknowledge that the lack of follow-up data limits our ability to further contextualize the differences between fulminant and non-fulminant myocarditis. This limitation has been added to the revised manuscript (lines 407-409). In future studies, we plan to conduct prospective research with systematic follow-up to better evaluate patient outcomes.

Revised text in Limitations: Lines 407-409: Second, the retrospective design limited data collection to available clinical records, and detailed information regarding in-hospital management, dynamic disease progression, and long-term follow-up was not consistently available.

10. **In the section on Limitations, on page 21 (lines 410-418), there is a discussion about "disease severity stage" which is confusing and unnecessary, and I would recommend omitting these sentences.**

Response: Thank you for this helpful suggestion. We agree that the discussion regarding “disease severity stage” was unclear and unnecessary. Accordingly, we have removed the original sentences.

**11. On Figure 2, a yellow bar noting no symptoms is missing.**

Response: Thank you for this suggestion. We have revised the manuscript. For completeness, “no symptoms” has been added to **S1 Fig**. The figure legend has been revised accordingly (lines 506-512).

Revised text: Lines 506-512: **S1 Fig.** Age-group distribution of clinical symptoms in children with fulminant myocarditis and acute non-fulminant myocarditis. The x-axis shows symptom categories, and the y-axis shows the percentage of patients in each age group with each symptom. Colors indicate different age groups. Percentages are calculated within each age group. Patients may have presented with multiple symptoms; therefore, percentages do not sum to 100%. One asymptomatic case was included (n = 1).

# Reviewer #2:

**Major Comments
1. Diagnostic Classification and Risk of Misclassification
The classification of fulminant myocarditis appears to rely primarily on clinical criteria without systematic confirmation using cardiac MRI or endomyocardial biopsy. While this approach is understandable in retrospective pediatric studies, it introduces a non-negligible risk of misclassification, particularly between severe acute non-fulminant myocarditis and fulminant myocarditis. This limitation should be more explicitly discussed, including its potential magnitude and its impact on predictor identification and the strength of reported associations.**

Response: Thank you for this important comment. We agree that the classification of FM in this study relied primarily on clinical criteria, without systematic confirmation by cardiac magnetic resonance (CMR) or endomyocardial biopsy (EMB) in all patients. Therefore, a non-negligible risk of misclassification may exist, particularly between severe ANFM and FM. We have also expanded the Methods (lines 119-123) and Discussion sections to address this limitation (lines 409-415).

Revised text: Lines 119-123: The revised text of methods section now reads: Based on published international and national guidelines and previous studies, FM was defined as acute myocarditis with rapid hemodynamic deterioration within 2 weeks of symptom onset, manifested as cardiogenic shock or life-threatening arrhythmias with hemodynamic instability, and requiring vasoactive agents and/or mechanical circulatory support (9, 12, 13).

Revised text in Limitations: Lines 409-415: Third, FM and ANFM were diagnosed primarily based on clinical grounds rather than systematic CMR or EMB confirmation, which may compromise diagnostic accuracy and introduce classification bias; variability in the timing of CMR can have a similar effect. Because FM classification relied mainly on rapidly progressive hemodynamic deterioration and the need for advanced life support, some degree of diagnostic misclassification between FM and severe ANFM cannot be excluded.

2. **Selection Bias
   The study was conducted in a single tertiary referral center, and patients transferred within 24 hours were included. This design may result in overrepresentation of more severe clinical phenotypes and patients requiring escalation of care. Consequently, both the observed prevalence of fulminant myocarditis and the strength of identified predictors may be influenced. This issue should be clearly acknowledged as a primary limitation of the study and discussed in terms of generalizability.**

Response: Thank you for this important comment. We agree that selection bias is a relevant concern in this study and have now explicitly acknowledged it as a major limitation in the revised manuscript. As a tertiary referral center with pediatric intensive care and advanced cardiopulmonary support, our institution receives a disproportionate number of severe myocarditis cases transferred from surrounding hospitals, which may have led to an overrepresentation of more severe clinical phenotypes in the cohort. Although non-fulminant myocarditis cases still accounted for the majority of the study population, this referral pattern may nevertheless have influenced both the observed prevalence of FM and the magnitude of some identified associations. We have therefore clarified in the Discussion section that the generalizability of our findings may be limited and further multicenter studies are needed (Lines 403-406).

Revised text in Limitations: Lines 403-406: First, as a single-center retrospective study conducted at a tertiary referral center, the findings may be subject to selection bias and limited generalizability, particularly given the relatively high proportion of severe myocarditis cases. External validation in multicenter cohorts is therefore needed.

3. **Model Stability and Overfitting Risk
   Although LASSO regression followed by multivariable logistic regression was applied, the number of fulminant myocarditis cases remains relatively limited (n=54). In addition, variable selection appears to be partially influenced by coefficient magnitude and clinical judgment. The absence of internal validation methods such as bootstrapping or cross-validation raises concerns regarding model stability and potential overfitting. Inclusion of internal validation analysis would substantially strengthen the robustness of the findings.**

Response: Thank you for this important comment. We have further standardized and clarified the variable selection process in the revised manuscript. First, candidate variables were selected using LASSO regression with 10-fold cross-validation, rather than being influenced by subjective clinical judgment. Given the limited number of events, the number of variables included in the final multivariable model was restricted according to the events-per-variable (EPV ≥ 10) principle to reduce the risk of overfitting. Among the variables identified by LASSO, predictors were prioritized based on their relative contribution (as reflected by the absolute magnitude of the coefficients), and the final set of variables was determined within the EPV constraint. Second, internal validation has been added using bootstrap resampling (1,000 iterations) to assess the stability and potential overfitting, and optimism-corrected performance metrics were reported. Relevant details have been incorporated into the Methods (lines 163-170, 176-180) and Results sections (lines 254-260).

Revised text in Methods: Lines 163-170: Variables were selected using LASSO regression with 10-fold cross-validation based on the λ.1se criterion. Given the limited number of events, the number of variables included in the final multivariable logistic regression was restricted according to the events-per-variable principle (EPV ≥ 10). Among variables selected by LASSO, indicators were prioritized based on their relative contribution (as reflected by the absolute magnitude of regression coefficients), and the final set of variables was determined within the EPV constraint.

Revised text in Methods: Lines 176-180: The discriminative ability of the selected indicators was evaluated using receiver operating characteristic (ROC) curves and the area under the curve (AUC). Calibration was assessed using calibration curves and the Hosmer–Lemeshow test. Internal validation was performed using bootstrap resampling (1,000 iterations) to obtain optimism-corrected AUC values.

Revised text in Results: Lines 254-260: The indicator combination showed good discriminatory ability, with an AUC of 0.935 (Fig 3). The optimal cutoff value was 0.435, with a sensitivity of 0.777, specificity of 0.941, positive predictive value of 0.893, and negative predictive value of 0.871. After bootstrap internal validation (B = 1,000), the optimism-corrected AUC was 0.918. Calibration curves showed good agreement between predicted and observed probabilities (Fig 4). The Hosmer–Lemeshow test indicated good fit (χ² = 2.469, *P* = 0.963).

### Fig 3. Receiver operating characteristic curve of the selected early warning indicator combination for fulminant myocarditis. Abbreviation: ROC, receiver operating characteristic curve.

Fig 4. Calibration curve of the combined early warning indicators for fulminant myocarditis.

4. **Lack of Predictive Performance Metrics
   The manuscript identifies independent predictors but does not report key model performance metrics such as: AUC / ROC curve analysis, Calibration measures, Sensitivity and specificity thresholds, Predictive values or risk stratification performance. These metrics are important for evaluating clinical applicability and translating the findings into clinical practice. Their inclusion would significantly improve the manuscript.**

Response: Thank you for this important comment. We have supplemented the revised manuscript with additional metrics, including ROC curves, AUC, sensitivity, specificity, positive predictive value, and negative predictive value. In addition, calibration curves and the Hosmer–Lemeshow test have been included in the Results section (lines 254-260).

Revised text: Lines 254-260: The indicator combination showed good discriminatory ability, with an AUC of 0.935 (Fig 3). The optimal cutoff value was 0.435, with a sensitivity of 0.777, specificity of 0.941, positive predictive value of 0.893, and negative predictive value of 0.871. After bootstrap internal validation (B = 1,000), the optimism-corrected AUC was 0.918. Calibration curves showed good agreement between predicted and observed probabilities (Fig 4). The Hosmer–Lemeshow test indicated good fit (χ² = 2.469, *P* = 0.963).

5. **Timing of Data Acquisition
   Clinical and laboratory variables were collected within a 24-hour window before or after admission. This may introduce heterogeneity, as some variables may reflect early inpatient evolution rather than initial emergency department presentation. Since the stated aim is to identify emergency department-based warning indicators, clarification of the timing of data collection relative to ED presentation is necessary.**

Response: Thank you for this insightful comment. As this study focused on early warning indicators of FM at the ED stage, all variables were intended to reflect information obtained at the time of first medical contact. However, we acknowledge that the timing of data collection relative to ED presentation was not clearly specified in the original manuscript. Therefore, we have further clarified this aspect in the Methods section (lines 138-143).

Revised text: Lines 138-143: All variables were derived from data obtained at the first medical encounter. For patients presented directly to our ED, the earliest available clinical and laboratory data were recorded. For transferred patients, data obtained at the referring hospital prior to transfer were prioritized. For examinations not completed during the ED visit (e.g.,echocardiography), the earliest available results on the day of admission were included.

**Minor Comments: The manuscript would benefit from professional language editing to correct grammatical and typographical errors and improve readability. Some sections of the abstract and discussion could be simplified for clarity. Tables are informative but could be reformatted to improve readability and clarity.**

Response: Thank you for this helpful comment. We have carefully revised the manuscript to improve grammar, wording, and overall readability. We also simplified several sentences in the abstract for greater clarity (lines 26-37). In addition, the tables have been reformatted to improve their presentation and readability (see Table 1 and Table 2).

Revised text in Abstract: Lines 26-37:

Methods
A retrospective analysis was conducted on 140 children with acute myocarditis admitted through the ED between 01/01/2015 and 31/12/2024. Patients were classified into acute non-fulminant myocarditis (ANFM) (n = 86) and FM (n = 54) groups. The least absolute shrinkage and selection operator (LASSO) regression was utilized to optimize variable selection, followed by multivariate logistic regression analysis to identify early warning indicators of FM.

## Results

Gastrointestinal symptoms were the most common clinical manifestation in children with FM (88.9%). Compared with the ANFM group, children with FM had lower serum sodium levels and higher lactate and myocardial injury marker levels (all *P* < 0.05).

# Reviewer #3:

1. **Issues Regarding Figure 1 Quality
   The vertical axis of Figure 1 lacks unit labels, preventing readers from determining whether the values represent the number of cases or percentages. Please clearly indicate the units in the axis label (e.g., "n" or "Number of patients"). Additionally, the font sizes and styles in the figure legend are inconsistent, which compromises the professionalism of the manuscript; please standardize all text fonts, sizes, and formatting in the legend to ensure compliance with the journal's requirements.
    Regarding the chart type, the manuscript currently uses a stacked bar chart to display symptom distribution across different age groups. However, the legend states that "Individual patients may have more than one symptom," meaning a single case could simultaneously belong to multiple symptom categories. This creates three significant problems: first, stacked bar charts inherently assume mutually exclusive categories (i.e., one case can only belong to one category), which contradicts the actual clinical scenario; second, readers cannot determine whether the bar height represents cumulative case counts or the incidence rate of individual symptoms; third, the chart may overestimate the total patient population, thereby compromising accurate data interpretation.
    We recommend replacing Figure 1 with a clustered bar chart (grouped bar chart), with symptom types on the horizontal axis (e.g., respiratory symptoms, gastrointestinal symptoms, cardiovascular symptoms, neurological symptoms) and incidence rates (percentages) for each age group on the vertical axis, using different colored bars to represent different age groups. This modification would more clearly display the incidence rates of various symptoms across age groups while avoiding misinterpretations resulting from case overlap.**

Response: Thank you for your valuable suggestion. We have revised the figure by replacing the stacked bar chart with a grouped bar chart, adding a y-axis label (“percentage of patients (%)”), and standardizing the font size and formatting. The revised figure clearly demonstrates the incidence rates of various symptoms across age groups (see S1 Fig).

To provide a more intuitive presentation of the age distribution between the two groups, we reconstructed Fig 1 as “Age distribution of children with fulminant myocarditis and acute non-fulminant myocarditis”. The distribution of clinical symptoms in the two groups has presented in Table 1. The original Fig 1 from the previous manuscript version has been moved to the Supporting Information as S1 Fig, where it serves as supplementary material to further illustrate the symptom distribution across different age groups. We made this adjustment to better align the focus of the manuscript with the exploration of early warning factors for FM.

Fig 1. Age distribution of children with fulminant myocarditis and acute non-fulminant myocarditis.

**S1 Fig.** Age-group distribution of clinical symptoms in children with fulminant myocarditis and acute non-fulminant myocarditis. The x-axis shows symptom categories, and the y-axis shows the percentage of patients in each age group with each symptom. Colors indicate different age groups. Percentages are calculated within each age group. Patients may have presented with multiple symptoms; therefore, percentages do not sum to 100%. One asymptomatic case was included (n = 1).

2. **Inconsistencies in Respiratory Symptom Terminology and Statistical Clarifications Needed
    2.1 In the first paragraph of the Results section, the authors state that "Shortness of breath/Tachypnea" occurred in 22.2% of cases in the FM group, whereas later the text describes that "Gastrointestinal symptoms were the most prevalent, occurring in 88.9% of cases." Although these figures are not contradictory per se, we recommend clarifying the relationship between "shortness of breath/tachypnea" and "dyspnea" when describing respiratory symptoms to avoid reader confusion.**

Response: Thank you for this helpful suggestion. We have revised the terminology throughout the manuscript to improve consistency and avoid potential confusion. In our study, tachypnea was used to indicate an increased respiratory rate, whereas dyspnea could include labored breathing, abnormal breath sounds, irregular respiratory rhythm, and cyanosis. To make this distinction clearer to readers, we have standardized the corresponding terminology in Table 1.

Revised text: Table 1: “Dyspnea and /or Tachypnea”

**2.2 Regarding electrocardiogram abnormalities, Table 2 shows that AVB occurred in 37.0% (20/54) of the FM group versus 5.8% (5/86) of the ANFM group (P<0.001); however, the Discussion claims that "AVB showed the strongest association with FM among all identified variables" and mentions that "Children presenting with AVB at ED admission had an approximately 11-fold increased risk of progression to FM." While this description is accurate (OR=10.890), we suggest adding a clarification in the Discussion that, although the absolute incidence of AVB was not the highest (being lower than the 88.9% for gastrointestinal symptoms), its association strength with FM (as reflected by the OR value) was indeed the strongest.**

Response: Thank you for this helpful suggestion. We agree that the absolute frequency of a variable and the strength of its association with FM are not the same. We have therefore revised the Discussion section to clarify that (lines 345-353).

Revised text: Lines 345-353: In our study, children presenting with third-degree AVB in the ED had approximately 32-fold higher odds of FM. Although the absolute incidence of third-degree AVB was not the highest (being lower than the 88.9% for gastrointestinal symptoms), its association with FM (as reflected by the OR value) was indeed the strongest. Previous studies have also reported that third-degree AVB is an important indicator of FM, allowing timely cardiac evaluation and therapeutic intervention (25). Given that electrocardiography is a readily available bedside examination in the ED, and that third-degree AVB showed a strong association with FM, it may serve as a key early warning indicator for the initial recognition of FM.

**2.3 Furthermore, regarding age distribution, the Results state that "The age of onset for the disease in the FM group predominantly occurred in preschool-aged children (3-7 years) and school-aged children (7-12 years)," yet Table 1 indicates that the median age in the FM group was 78 months (approximately 6.5 years, preschool age) versus 122 months (approximately 10 years) in the ANFM group. We recommend supplementing the text with specific age distribution data (such as exact numbers for each age subgroup) or providing an age distribution figure to substantiate the claim that the disease "mainly occurred in preschool and school-aged children."**

Response: Thank you for this suggestion. We have added a grouped bar chart to the main text showing the distribution of patient numbers across different age groups in the two groups, which more clearly presents the predominant age ranges in each group (Fig 1).

**2.4 Finally, concerning the selection of myocardial injury biomarkers, Table 2 demonstrates significant differences between groups for LDH, CK-MB, AST, and CK (all P<0.001) in addition to cTnI; however, only cTnI was selected in the final LASSO regression and multivariate analysis model. We suggest the authors add an explanation in the Discussion regarding why other myocardial injury biomarkers were excluded from the final early warning model—whether due to multicollinearity with cTnI or because of their lower predictive value.**

Response: Thank you for this helpful comment. In our study, key variables were primarily screened using LASSO regression, which helped address collinearity and reduce the risk of overfitting. CK was not carried forward beyond the univariable analysis. Although CK-MB and LDH were retained in the LASSO step, they were not included in the multivariate regression analysis because of the limited sample size and the prespecified events-per-variable principle, under which variables with lower relative contribution were excluded. We have clarified this point in the revised Discussion section (lines 327-329).

Revised text: Lines 327-329: Although CK-MB and LDH were initially retained in LASSO selection, they were excluded from the final multivariable analysis due to their relatively lower contribution under the prespecified EPV constraint.

3. **Discussion of Sex Differences
   Table 1 shows that males comprised 44.4% of the FM group compared with 76.7% of the ANFM group (P<0.001). This finding is particularly noteworthy because while myocarditis generally demonstrates male predominance, fulminant myocarditis appears more prevalent among females in this cohort. We recommend that the authors explore potential explanations for this unexpected sex distribution, discuss possible pathophysiological or immunological mechanisms, and cite relevant literature to support this observation.**

Response: Thank you for this helpful suggestion. We have added a brief discussion on sex-related differences in the Discussion section (lines 359-369).

Revised text: Lines 359-369: The higher proportion of males in the ANFM group may be related to sex hormone–mediated differences in immune responses, with androgens potentially promoting stronger pro-inflammatory responses and estrogens favoring anti-inflammatory and reparative processes (26, 27). Children with FM were also younger, which may reflect differences in clinical presentation. Younger children often rely on parental observation for non-specific symptoms, whereas older children can more clearly describe cardiovascular symptoms, enabling timely consultation. Although sex and age were selected by LASSO, they were not retained in the multivariable analysis. This may be due to their relatively lower contribution under the EPV constraint, the absence of significant sex differences within the FM group, and potential overlap between age and clinical presentation.

4. **Discussion of Age Factors
   Age represents one of the most significant differentiating variables between groups (P<0.001) and was selected in the LASSO regression analysis; however, it was not retained in the final multivariate model. We suggest the authors clarify in the Discussion: (1) the reasons why age did not enter the final predictive model; (2) whether the relationship between age and fulminant myocarditis is mediated by other variables (e.g., symptom types or specific biomarkers); and (3) whether age should be interpreted as a protective factor or a risk factor in this context.**

Response: Thank you for your valuable suggestion. In this study, children with FM were significantly younger than those with ANFM; however, age was not retained in the final multivariable analysis, likely due to its relatively lower contribution compared with other variables under the prespecified EPV constraint. This finding may reflect differences in clinical presentation across age groups. Younger children are more likely to present with non-specific symptoms, such as gastrointestinal manifestations (e.g., nausea, vomiting, or abdominal pain) and signs of hypoperfusion (e.g., pallor and fatigue), whereas older children more commonly present with cardiovascular symptoms and are better able to describe their complaints, potentially facilitating earlier medical attention. Therefore, age may be indirectly associated with FM through its influence on symptom presentation rather than acting as an independent risk or protective factor. We have clarified the role of age in the Discussion section (lines 359-369).

Revised text: Lines 359-369: The higher proportion of males in the ANFM group may be related to sex hormone–mediated differences in immune responses, with androgens potentially promoting stronger pro-inflammatory responses and estrogens favoring anti-inflammatory and reparative processes (26, 27). Children with FM were also younger, which may reflect differences in clinical presentation. Younger children often rely on parental observation for non-specific symptoms, whereas older children can more clearly describe cardiovascular symptoms, enabling timely consultation. Although sex and age were selected by LASSO, they were not retained in the multivariable analysis. This may be due to their relatively lower contribution under the EPV constraint, the absence of significant sex differences within the FM group, and potential overlap between age and clinical presentation.

5. **Clinical Significance of LVEF
   Table 2 demonstrates that the median left ventricular ejection fraction (LVEF) was significantly lower in the FM group (47.5%) compared with the ANFM group (64.1%) (P<0.001). However, the authors note that "owing to the retrospective design of this study, echocardiographic examinations were not consistently available at the time of ED presentation, leading to its exclusion from the early warning assessment." We recommend expanding the Discussion to address: (1) whether LVEF should be incorporated into the early warning model if echocardiographic data were consistently available at ED presentation; and (2) whether the other identified indicators in this study are sufficient to compensate for scenarios where immediate echocardiographic assessment cannot be obtained.**

Response: Thank you for this helpful suggestion. We performed separate LASSO analyses with and without LVEF. The results showed that when echocardiographic data are available at ED presentation, LVEF may improve the discriminatory performance of the early warning indicator combination. When echocardiography is not immediately available, the identified clinical and laboratory indicators still showed good discrimination and may support early risk stratification at initial presentation because they are readily obtainable. However, when echocardiographic data are available, these indicators should be considered complementary to, rather than a replacement for, echocardiographic assessment. We have revised the Methods, Results and Discussion section to clarify this.

Revised text in Methods: Lines 160-163: Echocardiography was not available for all patients at ED presentation, but LVEF was considered clinically important. Therefore, least absolute shrinkage and selection operator (LASSO) regression analyses were conducted both with and without LVEF.

Revised text in Results: Lines 254-262: The indicator combination showed good discriminatory ability, with an AUC of 0.935 (Fig 3). The optimal cutoff value was 0.435, with a sensitivity of 0.777, specificity of 0.941, positive predictive value of 0.893, and negative predictive value of 0.871. After bootstrap internal validation (B = 1,000), the optimism-corrected AUC was 0.918. Calibration curves showed good agreement between predicted and observed probabilities (Fig 4). The Hosmer–Lemeshow test indicated good fit (χ² = 2.469, *P* = 0.963). In a supplementary analysis including LVEF when available (S1 Table), the AUC increased to 0.973, indicating improved discriminaton (S2 Fig).

### Fig 3. Receiver operating characteristic curve of the selected early warning indicator combination for fulminant myocarditis. Abbreviation: ROC, receiver operating characteristic curve.

Fig 4. Calibration curve of the combined early warning indicators for fulminant myocarditis.

**S1 Table. Multivariable logistic regression analysis including left ventricular ejection fraction**

| **Variable** | **Coefficient** | **OR** | **95% CI** | ***P* value** |
| --- | --- | --- | --- | --- |
| Gastrointestinal symptoms | 2.176 | 8.813 | 1.701-45.646 | 0.010 |
| LVEF | -0.188 | 0.828 | 0.765-0.897 | <0.001 |
| Age | -0.034 | 0.966 | 0.946-0.987 | 0.002 |
| Third-degree AVB | 3.829 | 46.004 | 4.517-468.524 | 0.001 |
| Na | -0.229 | 0.795 | 0.654-0.968 | 0.022 |
| Abbreviation: LVEF, left ventricular ejection fraction; AVB, atrioventricular block; Na, serum sodium; OR, odds ratio; CI, confidence interval. | | | | |

### **S2 Fig.** Receiver operating characteristic curve of the supplementary analysis including left ventricular ejection fraction. Abbreviation: ROC, receiver operating characteristic curve.

Revised text in Discussion: Lines 386-400: Echocardiography is a first-line tool for assessing cardiac structure and function in children with suspected myocarditis. However, due to the retrospective study design and limited availability, echocardiographic data were often not available at ED presentation. Therefore, this study focused on exploring early warning indicators that do not rely on echocardiography. Nevertheless, echocardiography findings remain clinically important. In our cohort, LVEF was significantly lower in the FM group, with a median value below 50%, suggesting that many children with FM had already developed substantial cardiac dysfunction at presentation. Previous studies have shown that reduced LVEF is associated with the need for intensive care (18) and predicts a fulminant disease course (29). In supplementary analysis, inclusion of LVEF improved the discriminatory performance of early FM identification. However, even without LVEF, the combination of clinical and laboratory indicators still showed acceptable discrimination, supporting its practical value in the ED setting. In practice, early integration of laboratory findings and timely use of echocardiography, when available, may further improve early recognition of FM.

6. **Predictive Model Performance Evaluation
   Although this study identified five independent early warning indicators, no model performance metrics were provided, including sensitivity, specificity, receiver operating characteristic (ROC) curves with area under the curve (AUC) values, negative predictive value (NPV), or positive predictive value (PPV). We strongly recommend supplementing the manuscript with these essential evaluation metrics to enable readers to assess the clinical utility and diagnostic accuracy of this early warning model.**

Response: Thank you for this helpful comment. In the revised manuscript, we have added the AUC, sensitivity, specificity, PPV, and NPV to describe the discriminatory performance of the identified indicators. We also clarify that the aim of this study was to explore early warning indicators of FM rather than to develop a formal prediction model. Accordingly, these metrics are presented to show the discriminatory ability of the indicator combination. These revisions have been incorporated into the Methods (lines 176-180) and Results sections (lines 254-260).

Revised text in Methods: Lines 176-180: The discriminative ability of the selected indicators was evaluated using receiver operating characteristic (ROC) curves and the area under the curve (AUC). Calibration was assessed using calibration curves and the Hosmer–Lemeshow test. Internal validation was performed using bootstrap resampling (1,000 iterations) to obtain optimism-corrected AUC values.

Revised text in Results: Lines 254-260: The indicator combination showed good discriminatory ability, with an AUC of 0.935 (Fig 3). The optimal cutoff value was 0.435, with a sensitivity of 0.777, specificity of 0.941, positive predictive value of 0.893, and negative predictive value of 0.871. After bootstrap internal validation (B = 1,000), the optimism-corrected AUC was 0.918. Calibration curves showed good agreement between predicted and observed probabilities (Fig 4). The Hosmer–Lemeshow test indicated good fit (χ² = 2.469, *P* = 0.963).

7. **Discussion of External Validation
   While the authors acknowledge the limitation of single-center design, the Discussion inadequately addresses the critical importance of external validation. We suggest expanding this section to discuss: (1) specific strategies for conducting future external validation studies; (2) the necessity of multi-center collaborations to verify these findings; and (3) the potential applicability and generalizability of this early warning model across different ethnic populations, age distributions, and healthcare settings with varying resource availability.**

Response：Thank you for this helpful suggestion. We agree that the importance of external validation was not sufficiently discussed in the original manuscript. We have therefore expanded this point in the revised Discussion (lines 402-423).

Revised text in Discussion: Lines 402-423: First, as a single-center retrospective study conducted at a tertiary referral center, the findings may be subject to selection bias and limited generalizability, particularly given the relatively high proportion of severe myocarditis cases. External validation in multicenter cohorts is therefore needed.……Future prospective multicenter studies are needed to validate the robustness and generalizability of these findings across diverse populations, age groups, and healthcare settings.

8. **Discussion of Patient Outcomes
   Although the primary study outcome focused on identifying early warning indicators for FM progression, the manuscript did not report final patient prognoses, including mortality rates, ECMO utilization, or length of intensive care unit/hospital stay. We recommend supplementing the Discussion with: (1) mortality rates or incidence of severe complications in both the FM and ANFM groups; (2) associations between the identified early warning indicators and patient outcomes (e.g., whether elevated lactate levels correlate with increased mortality or ECMO requirement); and (3) the potential clinical value of early FM identification for improving patient prognosis and guiding resource allocation.**

Response: Thank you for this helpful suggestion. We agree that patient outcomes were insufficiently reported in the original manuscript. In the revised manuscript, we have added in-hospital outcome data, including mortality, ECMO use, and hospital stay to the Results section (lines 202-205). We have also expanded the discussion to address the potential associations between the identified early warning indicators and adverse outcomes (lines 298-304, lines 383-386), based on previous studies, and to highlight the potential clinical value of early FM identification for improving patient prognosis and guiding resource allocation.

Revised text in Results: Lines 202-205: Compared with the ANFM group, children with FM had a longer hospital stay (15.5 days vs. 7 days, *P* < 0.001), a higher rate of ECMO use (35.2% vs. 0.0%, *P* < 0.001), and higher mortality (9.3% vs. 0.0%, P=0.007) (Table 1).

Revised text in Discussion: Lines 298-304: A nationwide cohort study in Japan reported that Lac ≥2 mmol/L at admission was associated with a higher likelihood of receiving venoarterial extracorporeal membrane oxygenation (VA-ECMO)(15). Additionally, a multicenter retrospective study by Nichol *et al*. demonstrated that hyperlactatemia was independently associated with increased mortality among critically ill patients (16). These findings suggest that elevated Lac may reflect greater disease severity and poorer clinical outcomes.

Lines 383-386: By facilitating early identification of children at high risk for FM, our findings may assist emergency physicians in making more timely decisions regarding closer monitoring, early escalation of care, or the arrangement of early referral to tertiary centers with advanced circulatory support when appropriate.

9. **Sample Size Calculation
   The manuscript does not provide a sample size calculation or statistical power analysis. We recommend that the authors supplement this information by describing the study's statistical power, or alternatively, discuss whether the current sample size (54 FM and 86 ANFM cases) is adequate to detect the expected effect sizes with sufficient precision.**

Response: Thank you for this important comment. We agree that the original manuscript does not provide a sample size calculation or statistical power analysis. However, the final multivariable analysis was restricted according to the prespecified EPV principle. With 54 FM events and 5 retained variables, the EPV was 10.8, suggesting that the sample size was acceptable for this exploratory analysis. In addition, we performed 10-fold cross-validation during the LASSO procedure and bootstrap internal validation for the final indicator combination to further assess its stability. Nevertheless, the sample size remained limited, as reflected by the wide confidence intervals for some estimates, and the findings should therefore be interpreted cautiously and confirmed in larger external cohorts. We have clarified this point in the revised Discussion section (lines 415-424).

Revised text in Limitatons: Lines 415-424: Fourth, although the final analysis was restricted according to the prespecified EPV principle, the number of FM cases remained limited and no exernal validation was performed. ……Future prospective multicenter studies are needed to validate the robustness and generalizability of these findings across Future prospective multicenter studies are needed to validate the robustness and generalizability of these findings across diverse populations, age groups, and healthcare settings.

10. **Missing Data Management
    While the manuscript states that "Missing data were addressed using multiple imputation," it lacks details regarding which specific variables contained missing data, the proportion of missingness for each variable, and the particular methodology employed for the multiple imputation procedure (e.g., number of imputations, imputation model specifications). We recommend providing these technical details to enhance the transparency and reproducibility of the study.**

Response: Thank you for this helpful comment. We apologize for the lack of clarity regarding missing-data handling in the original manuscript. Among the candidate variables included in the main regression analyses, missing data were minimal, with all variables having less than 5% missingness. Specifically, only a few analyzed variables had missing values (e.g. convulsions (0.71%), CK-MB (0.71%), AST (0.71%), ECMO (1.43%), and LVEF (2.14%)), whereas the remaining variables were complete. Given this low level of missingness, simple imputation was considered appropriate. Missing values were imputed using the median for continuous variables and the mode for categorical variables. This approach has been clarified in the Methods section (lines 156-158). These revisions do not materially affect the main results.

Revised text in Methods: Lines 156-158: Given the low proportion of missing data (<5%), missing values were imputed using the median for continuous variables and the mode for categorical variables.

11. **Spelling and Grammatical Errors
    Several spelling and typographical errors are present throughout the manuscript. We recommend comprehensive proofreading of the entire text, with specific attention to the following corrections: In the Abstract, "Acut non-fulminant myocarditis" should be corrected to "Acute non-fulminant myocarditis"; In the Abstract, "emergency departemnt" should be corrected to "emergency department"; In the Keywords section, "Acut non-fulminant myocarditis" should be corrected to "Acute non-fulminant myocarditis"; In the Table 1 header, "IQR, interquartile rangen" should be corrected to "IQR, interquartile range".**

Response: Thank you for this helpful suggestion. We have carefully revised the manuscript to correct the relevant spelling and typographical errors.

# Reviewer #4:

**Major concerns:
1. The study includes both patients who presented directly to the authors’ emergency department and those who were transferred from other hospitals to the ED prior to admission. This raises important concerns regarding the validity of the “ED-based” predictors. For transferred patients, the initial clinical presentation and laboratory values would have been obtained at the referring institution, not at the study ED. Consequently, the variables used in the analysis may reflect a later stage of disease progression, rather than the true initial ED presentation. This undermines the premise that the identified indicators are early warning signs available at first medical contact. Furthermore, management prior to transfer (e.g., fluids, vasoactive support, ventilation) may have significantly altered laboratory and clinical parameters. Specify whether the analyzed variables represent first medical contact values, or values obtained after transfer.**

Response: Thank you for this valuable comment. This study focused on early warning indicators of FM at the ED stage. All variables were derived from data obtained at the first medical encounter. However, the timing of data collection relative to ED presentation was not clearly specified in the original manuscript. We have clarified this in the revised Methods section (lines 137-143).

Revised text: Lines 137-143: Candidate variables were selected based on clinical relevance, prior literature, and availability at initial medical contact. All variables were derived from data obtained at the first medical encounter. For patients presented directly to our ED, the earliest available clinical and laboratory data were recorded. For transferred patients, data obtained at the referring hospital prior to transfer were prioritized. For examinations not completed during the ED visit (e.g.,echocardiography), the earliest available results on the day of admission were included.

**2. the definition of FM is vague “On the basis of a diagnosis of acute myocarditis, patients who developed severe hemodynamic deterioration, cardiogenic shock, or life-threatening arrhythmias within a short period and required vasoactive agents or mechanical circulatory support were classified as FM”. The term “short period” is not defined, which introduces subjectivity and potential inconsistency in classification. In addition, life-threatening arrhythmias (ventricular tachycardia, high-grade AV block) can also occur in non-fulminant myocarditis, and are not specific to FM. The same goes for the need for intubation or circulatory/vasoactive support, which may also be observed in severe cases of non-fulminant myocarditis, particularly in tertiary care settings. As such, the current definition risks misclassification bias and may overlap significantly with markers of disease severity rather than representing a distinct clinical entity.
 In addition, because several predictors identified in the model (e.g., lactate, AV block) are themselves markers of hemodynamic compromise, there is a risk of incorporation bias, where predictors and outcome definitions are not fully independent.
 I suggest providing a clear and objective definition of “short period” (hours vs days from symptom onset or ED presentation), clarify whether FM classification was based on standardized criteria or institutional practice.**

Response: Thank you for this important comment. We agree that the definition of FM should be clearly specified to reduce potential subjectivity. In this study, FM was defined according to published international and national guidelines and previous studies, as acute myocarditis with rapid hemodynamic deterioration within a short petiond, and requiring inotropic support and/or mechanical circulatory support.

In the revised manuscript, we have clarified that “short period” refers to clinical deterioration occurring within 2 weeks of symptom onset. In our cohort, most cases progressing within a much shorter time frame (median 2 days) and all fatal cases deteriorated within 72 hours of admission. We also acknowledge that life-threatening arrhythmias and the need for ventilatory or circulatory support may occur in severe non-fulminant myocarditis. Therefore, FM classification in this study was based on the combination of rapid progression and acute hemodynamic collapse requiring support, rather than isolated clinical features. The definition used has been clarified in the revised Methods section (lines 119-123).

We agree that some identified indicators (e.g., lactate and high-grade atrioventricular block) are themselves markers of hemodynamic compromise at presentation and may therefore not be fully independent of the clinical definition of FM. As the primary aim of this study was to identify clinically useful early warning indicators at ED presentation rather than to establish independent causal predictors, variables reflecting early hemodynamic deterioration may still be of clinical value. We have revised the Discussion section to clarify this (lines 418-420).

Revised text in Methods: Lines 119-123: Based on published international and national guidelines and previous studies, FM was defined as acute myocarditis with rapid hemodynamic deterioration within 2 weeks of symptom onset, manifested as cardiogenic shock or life-threatening arrhythmias with hemodynamic instability, and requiring vasoactive agents and/or mechanical circulatory support (9, 12, 13).

Revised text in Limitations: Lines 418-420: Finally, some identified indicators, such as Lac and third-degree AVB, may partly reflect hemodynamic compromise, leading to potential incorporation bias that should be interpreted cautiously.

2. **Why was CMR not routinely performed?**

Response: Thank you for this comment. CMR was not routinely performed due to the retrospective design and the long study period, during which its availability and clinical use were limited, particularly in earlier years. In addition, the relatively long acquisition time and the need for patient cooperation may have restricted its use in children, especially in younger or hemodynamically unstable patients. This limitation has been acknowledged in the revised Discussion (lines 409-413).

Revised text in Limitations: Lines 409-413: Third, FM and ANFM were diagnosed primarily based on clinical grounds rather than systematic CMR or EMB confirmation, which may compromise diagnostic accuracy and introduce classification bias; variability in the timing of CMR can have a similar effect.

**3. Important clinical variables (e.g., blood pressure, LVEF, shock status) were excluded. While the authors justify this, these factors are critical determinants of severity and may confound associations. Either include these variables or clearly emphasize that the model reflects early risk markers rather than independent predictors.**

Response: Thank you for this valuable suggestion. We acknowledge the importance of key clinical variables such as LVEF and hemodynamic status. However, the primary aim of this study was to identify early warning indicators that are readily available in the ED, rather than to develop an independent predictive model. Therefore, these variables were not included in the main analysis. Nevertheless, their potential clinical value cannot be overlooked. We therefore conducted an additional analysis incorporating LVEF, which showed a strong association with FM. These findings have been added to the revised Methods (lines 160-163), Results (lines 261-262) and Discussion (lines 354-358, lines 388-401).

Revised text in Methods: Lines 160-163: Echocardiography was not available for all patients at ED presentation, but LVEF was considered clinically important. Therefore, least absolute shrinkage and selection operator (LASSO) regression analyses were conducted both with and without LVEF.

Revised text in Results: Lines 261-262: In a supplementary analysis including LVEF when available (S1 Table), the AUC increased to 0.973, indicating improved discriminaton (S2 Fig).

**S1 Table. Multivariable logistic regression analysis including left ventricular ejection fraction**

| **Variable** | **Coefficient** | **OR** | **95% CI** | ***P* value** |
| --- | --- | --- | --- | --- |
| Gastrointestinal symptoms | 2.176 | 8.813 | 1.701-45.646 | 0.010 |
| LVEF | -0.188 | 0.828 | 0.765-0.897 | <0.001 |
| Age | -0.034 | 0.966 | 0.946-0.987 | 0.002 |
| Third-degree AVB | 3.829 | 46.004 | 4.517-468.524 | 0.001 |
| Na | -0.229 | 0.795 | 0.654-0.968 | 0.022 |
| Abbreviation: LVEF, left ventricular ejection fraction; AVB, atrioventricular block; Na, serum sodium; OR, odds ratio; CI, confidence interval. | | | | |

### **S2 Fig.** Receiver operating characteristic curve of the supplementary analysis including left ventricular ejection fraction. Abbreviation: ROC, receiver operating characteristic curve.

Revised text in Discussion: Lines 354-358: FM may present with cardiogenic shock, and hypotension often draws immediate clinical attention. However, our study focused on early warning indicators preceding overt circulatory failure rather than on constructing a predictive model. Consequently, hypotension was not included in the analysis, although children with cardiogenic shock or low cardiac output were not excluded.

Revised text in Discussion: Lines 388-401: However, due to the retrospective study design and limited availability, echocardiographic data were often not available at ED presentation. Therefore, this study focused on exploring early warning indicators that do not rely on echocardiography. Nevertheless, echocardiography findings remain clinically important. In our cohort, LVEF was significantly lower in the FM group, with a median value below 50%, suggesting that many children with FM had already developed substantial cardiac dysfunction at presentation. Previous studies have shown that reduced LVEF is associated with the need for intensive care (18) and predicts a fulminant disease course (29). In supplementary analysis, inclusion of LVEF improved the discriminatory performance of early FM identification. However, even without LVEF, the combination of clinical and laboratory indicators still showed acceptable discrimination, supporting its practical value in the ED setting. In practice, early integration of laboratory findings and timely use of echocardiography, when available, may further improve early recognition of FM.

**4. While predictors are identified, the study does not provide Risk thresholds, Cut-off values, or a clinical scoring system. This limits translation into clinical practice. Please provide ROC analysis with optimal cutoffs if possible. Consider developing a simple clinical risk score.**

Response: Thank you for this helpful comment. In the revised manuscript, we have added the AUC, sensitivity, specificity, PPV, and NPV to describe the discriminatory performance of the identified indicators. As this study was exploratory and aimed to identify early warning indicators rather than to develop a formal prediction model, we did not construct a clinical risk score. Prospective multicenter studies are needed to develop and validate a clinically applicable risk score. We have revised the Methods (lines 176-180) and Results (lines 254-260) section.

Revised text in Methods: Lines 176-180: The discriminative ability of the selected indicators was evaluated using receiver operating characteristic (ROC) curves and the area under the curve (AUC). Calibration was assessed using calibration curves and the Hosmer–Lemeshow test. Internal validation was performed using bootstrap resampling (1,000 iterations) to obtain optimism-corrected AUC values.

Revised text in Results: Lines 254-260: The indicator combination showed good discriminatory ability, with an AUC of 0.935 (Fig 3). The optimal cutoff value was 0.435, with a sensitivity of 0.777, specificity of 0.941, positive predictive value of 0.893, and negative predictive value of 0.871. After bootstrap internal validation (B = 1,000), the optimism-corrected AUC was 0.918. Calibration curves showed good agreement between predicted and observed probabilities (Fig 4). The Hosmer–Lemeshow test indicated good fit (χ² = 2.469, *P* = 0.963).

### Fig 3. Receiver operating characteristic curve of the selected early warning indicator combination for fulminant myocarditis. Abbreviation: ROC, receiver operating characteristic curve.

Fig 4. Calibration curve of the combined early warning indicators for fulminant myocarditis.

**Minor comments:
1. Language and grammar: Multiple typographical errors (e.g., “departemnt”, “Acut non-fulminantmyocarditis”).**

Response: Thank you for the correction. We feel sorry for our carelessness. We have carefully revised the manuscript to correct typographical, spelling, and grammatical errors throughout the text.

**2. Timing of data collection: Some variables were collected within 24 hours after admission, which may not reflect true ED presentation variables. Clarify timing and restrict to ED data if possible.**

Response: Thank you for this important comment. We agree that the timing of data collection should be clearly specified. In this study, most clinical features, electrocardiographic findings, and laboratory variables were obtained at the initial ED evaluation or from the earliest available records at first medical contact. Echocardiographic data were often obtained shortly after admission, using the earliest available results. To address this, the primary analysis focused on variables readily available at ED presentation, while analyses including LVEF were performed as supplementary analyses. We acknowledge that, due to the retrospective design, a small proportion of variables may have been derived from the earliest available inpatient records. This has been clarified in the revised Methods (lines 138-143, lines 160-163) and Results sections (lines 261-262).

Revised text in Methods: Lines 138-143: All variables were derived from data obtained at the first medical encounter. For patients presented directly to our ED, the earliest available clinical and laboratory data were recorded. For transferred patients, data obtained at the referring hospital prior to transfer were prioritized. For examinations not completed during the ED visit (e.g.,echocardiography), the earliest available results on the day of admission were included.

Lines 160-163: Echocardiography was not available for all patients at ED presentation, but LVEF was considered clinically important. Therefore, least absolute shrinkage and selection operator (LASSO) regression analyses were conducted both with and without LVEF.

Revised text in Results: Lines 261-262: In a supplementary analysis including LVEF when available (S1 Table), the AUC increased to 0.973, indicating improved discriminaton (S2 Fig).

3. **ECG findings: AV block is grouped together; consider stratifying by degree.**

Response: Thank you very much for this valuable suggestion. In response to your comment, we further categorized AVB by degree (first-, second-, and third-degree AVB) and added the comparison of different AVB grades between the FM and non-FM groups in the revised manuscript (see Table 2). The results showed that third-degree AVB was substantially more frequent in the FM group than in the ANFM group. Based on this finding, third-degree AVB was entered as a variable in the LASSO regression and multivariable logistic regression analyses, where it remained significantly associated with FM (Results: lines 248-252). We have also revised the Discussion to emphasize its potential value as an early warning sign of FM (lines 345-353).

Table 2：

| **Variable** | **FM (n=54)** | **ANFM (n=86)** | ***P* Value** |
| --- | --- | --- | --- |
| First-degree AVB | 4 (7.4) | 2(2.3) | 0.205 |
| Second-degree AVB | 1 (1.9) | 1 (1.2) | 0.624 |
| Third-degree AVB | 15 (27.8) | 2 (2.3) | <0.001 |

### Revised text in Results: Lines 248-252: Ultimately, five warning indicators of FM were identified (Table 3): gastrointestinal symptoms (OR = 11.468, 95% CI: 2.495–52.713, *P* = 0.002), Lac (OR = 1.887, 95% CI: 1.155–3.082, *P* = 0.011), cTnI (OR = 1.188, 95% CI: 1.055–1.337, *P* = 0.004), third-degree AVB (OR = 32.211, 95% CI: 2.943–352.484, *P* = 0.004), and Na (OR = 0.785, 95% CI: 0.652–0.944, *P* = 0.010)（Table 3).

Revised text in Discussion: Lines 345-353: In our study, children with third-degree AVB in the ED had approximately 32-fold higher odds of FM. Although the absolute incidence of third-degree AVB was not the highest (being lower than the 88.9% for gastrointestinal symptoms), its association strength with FM (as reflected by the OR value) was indeed the strongest. Previous studies have also reported that third-degree AVB is an important indicator of FM, allowing timely cardiac evaluation and therapeutic intervention (25). Given that electrocardiography is a readily available bedside examination in the ED, and that third-degree AVB showed a strong association with FM, it may serve as a key early warning indicator for the initial recognition of FM.

**4. Troponin interpretation OR per unit increase is difficult to interpret clinically; consider transformation or clinically meaningful increments.**

Response: Thank you for this helpful comment. We agree that interpretation of the odds ratio for cTnI per unit increase may be less intuitive clinically. In the revised Discussion (lines 321-327), we have revised the text to emphasize that higher cTnI levels were independently associated with FM, while avoiding over-interpretation of the per-unit effect estimate.

Revised text in Discussion: Lines 321-327: In our study, initial cTnI levels were significantly higher in children with FM, consistent with findings from a nationwide Japanese study (2), suggesting its potential value for early risk stratification at ED presentation. However, previous studies have shown that cTnI may also be markedly elevated in children with only mild ventricular dysfunction and do not always correlate linearly with disease severity (17-19). Therefor, cTnI should be interpreted in combination with other indicators.

**5. Figures: Ensure high resolution and improved clarity of LASSO plots.**

Response: Thank you for this helpful comment. The LASSO plots have been revised to improve clarity and resolution. The Fig 2 has been exported at higher resolution (600 dpi) to meet journal requirements.

**6. Abbreviations: Standardize terminology (e.g., Lac vs lactate, cTnI formatting).**

Response: Thank you for this suggestion. We have standardized the terminology and abbreviations throughout the manuscript. In particular, inconsistent expressions (e.g., “Lac” vs “lactate” and formatting of “cTnI”) have been unified to ensure clarity and consistency.

# **Reviewer #5:**

**Early detection in practice remains challenging due to the nonspecific nature of early clinical manifestations, and the work does not clearly articulate the potential clinical impact of the findings: morbidity or mortality; apart from its prediction for fulminant cases.**

Response: Thank you for this important comment. We agree that early detection of FM is challenging because the initial clinical manifestations are often nonspecific. In the revised Discussion (lines 370-386), we have clarified that early identification may support timely management decisions and improve patient outcomes.

Revised text in Discussion: Lines 370-386: Early identification of FM may help facilitate timely targeted treatment, improve the likelihood of successful rescue, and reduce the risk of mortality (13).……By facilitating early identification of children at high risk for FM, our findings may assist emergency physicians in making more timely decisions regarding closer monitoring, early escalation of care, or the arrangement of early referral to tertiary centers with advanced circulatory support when appropriate.

**Furthermore, the relative importance of the identified predictors is not well established, as no incremental values or comparisons among these factors are provided.**

Response: Thank you for this important comment. We agree that the relative importance of the identified indicators was not formally compared in the original manuscript. However, the primary aim of this study was to identify clinically useful early warning indicators at ED presentation rather than to establish a weighted prediction model. This may be an important direction for future prospective studies. Nevertheless, some variables, such as third-degree AVB, showed particularly strong associations with FM in our cohort and may represent important clinical warning signals. We have added a brief clarification in the Discussion to reflect this point (lines 345-353).

Revised text in Discussion: Lines 345-353: In our study, children with third-degree AVB in the ED had approximately 32-fold higher odds of FM. Although the absolute incidence of third-degree AVB was not the highest (being lower than the 88.9% for gastrointestinal symptoms), its association with FM (as reflected by the OR value) was indeed the strongest. ……Given that electrocardiography is a readily available bedside examination in the ED, and that third-degree AVB showed a strong association with FM, it may serve as a key early warning indicator for the initial recognition of FM.

**The applicability of the results is slightly overstated, and the conclusions would be strengthened by more cautious language—such as indicating that these factors "may help identify high-risk patients"—and by emphasizing the need for validation in prospective, multicenter studies.**

Response: Thank you for this helpful suggestion. We agree that the applicability of the findings should not be overstated. We have therefore revised the Conclusions to use more cautious language and to highlight the need for validation in prospective, multicenter studies (426-429).

# Revised text in Conclusions: Lines 426-429: Elevated Lac and cTnI levels, decreased Na levels, gastrointestinal symptoms, and third-degree AVB were associated with FM at ED presentation. These readily available indicators may facilitate early recognition of FM in the emergency setting. Prospective multicenter studies are needed to validate these findings.
