# Supplementary material for: Clinical characteristics and emergency department-based warning indicators of fulminant myocarditis in children
Source: PLoS One. 2026 Sep 17;21(9):e0358335. doi: 10.1371/journal.pone.0358335 (PMC13585240; doi:10.1371/journal.pone.0358335)
Supplement: Supplementary file 7 [file pone.0358335.s007.docx]

Dear Editor and Reviewers,

We sincerely thank you for your valuable and insightful comments on our manuscript entitled “Clinical characteristics and emergency department-based warning indicators of fulminant myocarditis in children”. Your constructive comments have greatly improved the quality and clarity of our manuscript. We have carefully addressed all comments and revised the manuscript accordingly. Our point-by-point responses are provided below, and all revisions in the manuscript have been highlighted in blue.

# Reviewer #2:

# 1-Data availability statement inconsistency The submission form indicates that some restrictions apply to data availability, whereas the manuscript states that all relevant data are included in the paper and Supporting Information files, including the anonymized minimal dataset as S1 Data.xlsx. These statements should be made fully consistent. If the dataset is fully available, the submission form should reflect this. If restrictions apply, the nature of the restrictions should be explicitly stated.

Response: Thank you for this valuable suggestion. The Data Availability Statement has been updated to clarify that all relevant data are included in the manuscript and its Supporting Information files, including the anonymized minimal dataset as S1 Data.xlsx. We also confirmed with the PLOS Editorial Office that the outdated “restrictions” statement displayed in the generated PDF is being phased out by the journal and will not be considered during editorial or peer review.

2-**Residual language and typographical errors
  The manuscript still contains several typographical and grammatical errors that should be corrected before publication. Examples include:
  “abserved” should be “observed”
  “Therefor” should be “Therefore”
  “porgression” should be “progression”
  “exernal validation” should be “external validation”
  “discriminaton” should be “discrimination”
  spacing errors such as “(1).Pediatric” and “e.g.,echocardiography”
  inconsistent capitalization/formatting of cTnI/CTnI
  duplicated wording in the Limitations section: “Future prospective multicenter studies are needed to validate the robustness and generalizability of these findings across Future prospective multicenter studies…”
  the manuscript should undergo careful language editing.**

Response: Thank you for this valuable suggestion. We have carefully proofread the manuscript and corrected the identified typographical, grammatical, formatting, and language errors throughout the text. The revisions include corrections to terminology (e.g., multivariable), spacing and punctuation, formatting consistency (e.g., cTnI), grammatical errors, and removal of the duplicated sentence in the Limitations section. The specific revisions are listed below.

**Revised text:**

**Line 31:** multivariate → multivariable;

Line 67: (1).Pediatric → (1). Pediatric;

Line 74: (ANFM)(8) → (ANFM) (8);

Line 143: e.g.,echocardiography → e.g., echocardiography；

Line 150: electrocardiogram → electrocardiography;

Line 207: *P*=0.007 → *P* = 0.007;

Line 216: (all *P <* 0.05), (Table 2) → (all *P <* 0.05) (Table 2);

Line 224: (*P <* 0.05). (Table 2) → (*P <* 0.05) (Table 2);

Line 217: abserved → observed;

Line 244: ***λ* 1se** → ***λ.*1se;**

**Line 246:** ***λ* 1se** → ***λ.*1se;**

**Line 263:** discriminaton → discrimination;

Line 302: (VA-ECMO)(15) → (VA-ECMO) (15);

Line 327: Therefor → Therefore;

Line 333: mechanism → mechanisms;

Line 341: porgression → progression;

Line 379: discrimination → discriminative;

Line 419: exernal → external;

Table 2: CTnI → cTnI;

The duplicated sentence in the Limitations section was removed.

**3-Clarify terminology: warning indicators vs prediction model
The authors correctly state that this study identifies early warning indicators rather than developing a formal prediction model. This distinction should remain consistent throughout the manuscript. Terms such as “predictive model” or “model performance” should be used cautiously unless clearly referring only to the discriminatory performance of the indicator combination.**

**Response:** Thank you for this valuable suggestion. We have carefully reviewed the manuscript to ensure consistent use of terminology. The manuscript does not describe the development of a prediction model or refer to model performance. We have also revised the wording in several places to consistently emphasize that this study identifies **warning indicators** rather than developing a prediction model.

**Revised text:** Line 254: “indicator combination” was revised to “combination of warning indicators”;

**Line 397:** In the supplementary analysis, inclusion of LVEF improved the discriminative ability of the warning indicators for early FM identification.

**4-Interpretation of incorporation bias
The authors now acknowledge that lactate and third-degree AV block may partly reflect hemodynamic compromise and may overlap with the clinical definition of fulminant myocarditis. This limitation is appropriate and should remain clearly stated. The conclusions should continue to avoid implying causal or independently predictive effects.**

Response: Thank you for this valuable suggestion. The limitation regarding potential incorporation bias has been retained in the Limitations section, and the Conclusions use cautious wording to describe an association rather than causality or independent predictive effects.

5- **Minor table and figure formatting
   Tables and figures are generally informative, but final formatting should be checked for journal consistency, especially abbreviations, spacing, decimal formatting, and image resolution.**

Response: Thank you for this valuable suggestion. We have carefully reviewed and revised the formatting of all tables and figures to ensure consistency with the journal's requirements, including abbreviations, spacing, decimal formatting, and image resolution.
