# Supplementary material for: Clinical characteristics and emergency department-based warning indicators of fulminant myocarditis in children
Source: PLoS One. 2026 Sep 17;21(9):e0358335. doi: 10.1371/journal.pone.0358335 (PMC13585240; doi:10.1371/journal.pone.0358335)
Supplement: Peer review history

# Peer review: Decision Letter 0

**DOI:** 10.1371/journal.pone.0358335.r001

**Authors:** Hany Abo-Haded

9 Apr 2026

Clinical characteristics and emergency department-based warning indicators of fulminant myocarditis in children

PLOS One

Dear Dr. Song,

Thank you for submitting your manuscript to PLOS ONE. After careful consideration, we feel that it has merit but does not fully meet PLOS ONE’s publication criteria as it currently stands. Therefore, we invite you to submit a revised version of the manuscript that addresses the points raised during the review process.

Please submit your revised manuscript by May 24 2026 11:59PM. If you will need more time than this to complete your revisions, please reply to this message or contact the journal office at plosone@plos.org. When you're ready to submit your revision, log on to https://www.editorialmanager.com/pone/ and select the 'Submissions Needing Revision' folder to locate your manuscript file.

- A letter that responds to each point raised by the academic editor and reviewer(s). You should upload this letter as a separate file labeled 'Response to Reviewers'.
- A marked-up copy of your manuscript that highlights changes made to the original version. You should upload this as a separate file labeled 'Revised Manuscript with Track Changes'.
- An unmarked version of your revised paper without tracked changes. You should upload this as a separate file labeled 'Manuscript'.

If you would like to make changes to your financial disclosure, please include your updated statement in your cover letter. Guidelines for resubmitting your figure files are available below the reviewer comments at the end of this letter.

If applicable, we recommend that you deposit your laboratory protocols in protocols.io to enhance the reproducibility of your results. Protocols.io assigns your protocol its own identifier (DOI) so that it can be cited independently in the future. For instructions see: https://journals.plos.org/plosone/s/submission-guidelines#loc-laboratory-protocols. Additionally, PLOS ONE offers an option for publishing peer-reviewed Lab Protocol articles, which describe protocols hosted on protocols.io. Read more information on sharing protocols at https://plos.org/protocols?utm_medium=editorial-email&utm_source=authorletters&utm_campaign=protocols.

As the corresponding author, your ORCID iD is verified in the submission system and will appear in the published article. PLOS supports the use of ORCID, and we encourage all coauthors to register for an ORCID iD and use it as well. Please encourage your coauthors to verify their ORCID iD within the submission system before final acceptance, as unverified ORCID iDs will not appear in the published article. *Only*  the individual author can complete the verification step; PLOS staff *cannot*  verify ORCID iDs on behalf of authors.

We look forward to receiving your revised manuscript.

Kind regards,

Hany Mahmoud Abo-Haded, MD

Academic Editor

PLOS One

Journal Requirements:

When submitting your revision, we need you to address these additional requirements.

1. Please ensure that your manuscript meets PLOS ONE's style requirements, including those for file naming. The PLOS ONE style templates can be found at

https://journals.plos.org/plosone/s/file?id=wjVg/PLOSOne_formatting_sample_main_body.pdf and https://journals.plos.org/plosone/s/file?id=ba62/PLOSOne_formatting_sample_title_authors_affiliations.pdf

2. We note that you have indicated that there are restrictions to data sharing for this study. For studies involving human research participant data or other sensitive data, we encourage authors to share de-identified or anonymized data. However, when data cannot be publicly shared for ethical reasons, we allow authors to make their data sets available upon request. For information on unacceptable data access restrictions, please see http://journals.plos.org/plosone/s/data-availability#loc-unacceptable-data-access-restrictions.

Before we proceed with your manuscript, please address the following prompts:

a) If there are ethical or legal restrictions on sharing a de-identified data set, please explain them in detail (e.g., data contain potentially identifying or sensitive patient information, data are owned by a third-party organization, etc.) and who has imposed them (e.g., a Research Ethics Committee or Institutional Review Board, etc.). Please also provide contact information for a data access committee, ethics committee, or other institutional body to which data requests may be sent.

b) If there are no restrictions, please upload the minimal anonymized data set necessary to replicate your study findings to a stable, public repository and provide us with the relevant URLs, DOIs, or accession numbers. Please see http://www.bmj.com/content/340/bmj.c181.long for guidelines on how to de-identify and prepare clinical data for publication. For a list of recommended repositories, please see https://journals.plos.org/plosone/s/recommended-repositories. You also have the option of uploading the data as Supporting Information files, but we would recommend depositing data directly to a data repository if possible.

Please update your Data Availability statement in the submission form accordingly.

3. PLOS requires an ORCID iD for the corresponding author in Editorial Manager on papers submitted after December 6th, 2016. Please ensure that you have an ORCID iD and that it is validated in Editorial Manager. To do this, go to ‘Update my Information’ (in the upper left-hand corner of the main menu), and click on the Fetch/Validate link next to the ORCID field. This will take you to the ORCID site and allow you to create a new iD or authenticate a pre-existing iD in Editorial Manager.

If the reviewer comments include a recommendation to cite specific previously published works, please review and evaluate these publications to determine whether they are relevant and should be cited. There is no requirement to cite these works unless the editor has indicated otherwise.

[Note: HTML markup is below. Please do not edit.]

Reviewers' comments:

Reviewer's Responses to Questions

**Comments to the Author**

1. Is the manuscript technically sound, and do the data support the conclusions?

Reviewer #1: Yes

Reviewer #2: Yes

Reviewer #3: Yes

Reviewer #4: Yes

Reviewer #5: Partly

**********

2. Has the statistical analysis been performed appropriately and rigorously?

Reviewer #1: Yes

Reviewer #2: No

Reviewer #3: Yes

Reviewer #4: Yes

Reviewer #5: Yes

**********

3. Have the authors made all data underlying the findings in their manuscript fully available?

The PLOS Data policy

Reviewer #1: Yes

Reviewer #2: No

Reviewer #3: No

Reviewer #4: No

Reviewer #5: Yes

**********

4. Is the manuscript presented in an intelligible fashion and written in standard English?

Reviewer #1: Yes

Reviewer #2: No

Reviewer #3: Yes

Reviewer #4: Yes

Reviewer #5: Yes

**********

Reviewer #1: 1. Well written manuscript with fairly large number of cases at a single pediatric ED.

2. Description of standard statistical analyses clear, but the LASSO method is not commonly seen in pediatric manuscripts, and I would think needs a fuller explanation for most readers.

3. A strength of the manuscript is the emphasis on how myocarditis (acute and non-acute) initially presents to an emergency room. But exclusion of cases that have obvious low output/shock symptoms skews the actual proportion of cases of myocarditis that would fall in the "fulminant category".

4. It is understandable that the number of patients with a CMR is limited in this retrospective review, but the fact that only 29 of 46 patients had a positive study confirming the diagnosis merits some discussion. Since a CMR is currently the most reliable diagnostic study (other than the relatively infrequently used biopsy), the accuracy of the diagnosis could be questioned. This concern would be mitigated by giving information on the timing of the CMR compared to ED presentation.

5. Highlighting the clinical and lab findings that help identify fulminant myocarditis is valuable. However, in the discussion of troponin (page 17, lines 324-341), it important to point out that very high levels can sometimes be found in non-fulminant myocarditis (emphasized in the Scientific Statement by Law, et al - reference 17).

6. Presenting symptoms considered should include syncope or near syncope (page 6, lines128-131).

7. The biomarker of BNP or pro-BNP may be useful at presentation, but it was not mentioned.

8. The importance of AV block is a key finding; but it needs to be broken down into the degrees of block.

9. There is no mention of the follow up of the patients in this series, both short and longer term follow if available would-be important information to put the diagnosis of fulminant and non-fulminant in perspective.

10. In the section on Limitations, on page 21 (lines 410-418), there is a discussion about "disease severity stage" which is confusing and unnecessary, and I would recommend omitting these sentences.

11. On Figure 2, a yellow bar noting no symptoms is missing.

Reviewer #2: This manuscript evaluates early warning indicators of fulminant myocarditis (FM) in children presenting to the emergency department using a retrospective cohort of 140 patients. The topic is clinically meaningful, particularly in the context of early risk stratification, escalation of care, and timely consideration of advanced circulatory support in pediatric myocarditis. The focus on readily available clinical and laboratory parameters enhances the potential clinical applicability of the study. The study benefits from a relatively large cohort for a single-center pediatric study and from the use of a structured statistical framework combining LASSO regression and multivariable logistic regression. However, several methodological limitations reduce the internal validity and translational impact of the findings.

Major Comments

1. Diagnostic Classification and Risk of Misclassification

The classification of fulminant myocarditis appears to rely primarily on clinical criteria without systematic confirmation using cardiac MRI or endomyocardial biopsy. While this approach is understandable in retrospective pediatric studies, it introduces a non-negligible risk of misclassification, particularly between severe acute non-fulminant myocarditis and fulminant myocarditis. This limitation should be more explicitly discussed, including its potential magnitude and its impact on predictor identification and the strength of reported associations.

2. Selection Bias

The study was conducted in a single tertiary referral center, and patients transferred within 24 hours were included. This design may result in overrepresentation of more severe clinical phenotypes and patients requiring escalation of care. Consequently, both the observed prevalence of fulminant myocarditis and the strength of identified predictors may be influenced. This issue should be clearly acknowledged as a primary limitation of the study and discussed in terms of generalizability.

3. Model Stability and Overfitting Risk

Although LASSO regression followed by multivariable logistic regression was applied, the number of fulminant myocarditis cases remains relatively limited (n=54). In addition, variable selection appears to be partially influenced by coefficient magnitude and clinical judgment. The absence of internal validation methods such as bootstrapping or cross-validation raises concerns regarding model stability and potential overfitting. Inclusion of internal validation analysis would substantially strengthen the robustness of the findings.

4. Lack of Predictive Performance Metrics

The manuscript identifies independent predictors but does not report key model performance metrics such as: AUC / ROC curve analysis, Calibration measures, Sensitivity and specificity thresholds, Predictive values or risk stratification performance. These metrics are important for evaluating clinical applicability and translating the findings into clinical practice. Their inclusion would significantly improve the manuscript.

5. Timing of Data Acquisition

Clinical and laboratory variables were collected within a 24-hour window before or after admission. This may introduce heterogeneity, as some variables may reflect early inpatient evolution rather than initial emergency department presentation. Since the stated aim is to identify emergency department-based warning indicators, clarification of the timing of data collection relative to ED presentation is necessary.

Minor Comments: The manuscript would benefit from professional language editing to correct grammatical and typographical errors and improve readability. Some sections of the abstract and discussion could be simplified for clarity. Tables are informative but could be reformatted to improve readability and clarity.

Conclusion: This study addresses an important clinical problem and provides potentially useful early indicators for fulminant myocarditis in children. However, the methodological limitations described above reduce the strength and generalizability of the conclusions.

Major revision is recommended before the manuscript can be considered for publication.

Reviewer #3: 1. Issues Regarding Figure 1 Quality

The vertical axis of Figure 1 lacks unit labels, preventing readers from determining whether the values represent the number of cases or percentages. Please clearly indicate the units in the axis label (e.g., "n" or "Number of patients"). Additionally, the font sizes and styles in the figure legend are inconsistent, which compromises the professionalism of the manuscript; please standardize all text fonts, sizes, and formatting in the legend to ensure compliance with the journal's requirements.

Regarding the chart type, the manuscript currently uses a stacked bar chart to display symptom distribution across different age groups. However, the legend states that "Individual patients may have more than one symptom," meaning a single case could simultaneously belong to multiple symptom categories. This creates three significant problems: first, stacked bar charts inherently assume mutually exclusive categories (i.e., one case can only belong to one category), which contradicts the actual clinical scenario; second, readers cannot determine whether the bar height represents cumulative case counts or the incidence rate of individual symptoms; third, the chart may overestimate the total patient population, thereby compromising accurate data interpretation.

We recommend replacing Figure 1 with a clustered bar chart (grouped bar chart), with symptom types on the horizontal axis (e.g., respiratory symptoms, gastrointestinal symptoms, cardiovascular symptoms, neurological symptoms) and incidence rates (percentages) for each age group on the vertical axis, using different colored bars to represent different age groups. This modification would more clearly display the incidence rates of various symptoms across age groups while avoiding misinterpretations resulting from case overlap.

2.Inconsistencies in Respiratory Symptom Terminology and Statistical Clarifications Needed

In the first paragraph of the Results section, the authors state that "Shortness of breath/Tachypnea" occurred in 22.2% of cases in the FM group, whereas later the text describes that "Gastrointestinal symptoms were the most prevalent, occurring in 88.9% of cases." Although these figures are not contradictory per se, we recommend clarifying the relationship between "shortness of breath/tachypnea" and "dyspnea" when describing respiratory symptoms to avoid reader confusion. Regarding electrocardiogram abnormalities, Table 2 shows that AVB occurred in 37.0% (20/54) of the FM group versus 5.8% (5/86) of the ANFM group (P<0.001); however, the Discussion claims that "AVB showed the strongest association with FM among all identified variables" and mentions that "Children presenting with AVB at ED admission had an approximately 11-fold increased risk of progression to FM." While this description is accurate (OR=10.890), we suggest adding a clarification in the Discussion that, although the absolute incidence of AVB was not the highest (being lower than the 88.9% for gastrointestinal symptoms), its association strength with FM (as reflected by the OR value) was indeed the strongest. Furthermore, regarding age distribution, the Results state that "The age of onset for the disease in the FM group predominantly occurred in preschool-aged children (3-7 years) and school-aged children (7-12 years)," yet Table 1 indicates that the median age in the FM group was 78 months (approximately 6.5 years, preschool age) versus 122 months (approximately 10 years) in the ANFM group. We recommend supplementing the text with specific age distribution data (such as exact numbers for each age subgroup) or providing an age distribution figure to substantiate the claim that the disease "mainly occurred in preschool and school-aged children." Finally, concerning the selection of myocardial injury biomarkers, Table 2 demonstrates significant differences between groups for LDH, CK-MB, AST, and CK (all P<0.001) in addition to cTnI; however, only cTnI was selected in the final LASSO regression and multivariate analysis model. We suggest the authors add an explanation in the Discussion regarding why other myocardial injury biomarkers were excluded from the final early warning model—whether due to multicollinearity with cTnI or because of their lower predictive value.

3.Discussion of Sex Differences

Table 1 shows that males comprised 44.4% of the FM group compared with 76.7% of the ANFM group (P<0.001). This finding is particularly noteworthy because while myocarditis generally demonstrates male predominance, fulminant myocarditis appears more prevalent among females in this cohort. We recommend that the authors explore potential explanations for this unexpected sex distribution, discuss possible pathophysiological or immunological mechanisms, and cite relevant literature to support this observation.

4. Discussion of Age Factors

Age represents one of the most significant differentiating variables between groups (P<0.001) and was selected in the LASSO regression analysis; however, it was not retained in the final multivariate model. We suggest the authors clarify in the Discussion: (1) the reasons why age did not enter the final predictive model; (2) whether the relationship between age and fulminant myocarditis is mediated by other variables (e.g., symptom types or specific biomarkers); and (3) whether age should be interpreted as a protective factor or a risk factor in this context.

5. Clinical Significance of LVEF

Table 2 demonstrates that the median left ventricular ejection fraction (LVEF) was significantly lower in the FM group (47.5%) compared with the ANFM group (64.1%) (P<0.001). However, the authors note that "owing to the retrospective design of this study, echocardiographic examinations were not consistently available at the time of ED presentation, leading to its exclusion from the early warning assessment." We recommend expanding the Discussion to address: (1) whether LVEF should be incorporated into the early warning model if echocardiographic data were consistently available at ED presentation; and (2) whether the other identified indicators in this study are sufficient to compensate for scenarios where immediate echocardiographic assessment cannot be obtained.

6. Predictive Model Performance Evaluation

Although this study identified five independent early warning indicators, no model performance metrics were provided, including sensitivity, specificity, receiver operating characteristic (ROC) curves with area under the curve (AUC) values, negative predictive value (NPV), or positive predictive value (PPV). We strongly recommend supplementing the manuscript with these essential evaluation metrics to enable readers to assess the clinical utility and diagnostic accuracy of this early warning model.

7. Discussion of External Validation

While the authors acknowledge the limitation of single-center design, the Discussion inadequately addresses the critical importance of external validation. We suggest expanding this section to discuss: (1) specific strategies for conducting future external validation studies; (2) the necessity of multi-center collaborations to verify these findings; and (3) the potential applicability and generalizability of this early warning model across different ethnic populations, age distributions, and healthcare settings with varying resource availability.

8. Discussion of Patient Outcomes

Although the primary study outcome focused on identifying early warning indicators for FM progression, the manuscript did not report final patient prognoses, including mortality rates, ECMO utilization, or length of intensive care unit/hospital stay. We recommend supplementing the Discussion with: (1) mortality rates or incidence of severe complications in both the FM and ANFM groups; (2) associations between the identified early warning indicators and patient outcomes (e.g., whether elevated lactate levels correlate with increased mortality or ECMO requirement); and (3) the potential clinical value of early FM identification for improving patient prognosis and guiding resource allocation.

9. Sample Size Calculation

The manuscript does not provide a sample size calculation or statistical power analysis. We recommend that the authors supplement this information by describing the study's statistical power, or alternatively, discuss whether the current sample size (54 FM and 86 ANFM cases) is adequate to detect the expected effect sizes with sufficient precision.

10. Missing Data Management

While the manuscript states that "Missing data were addressed using multiple imputation," it lacks details regarding which specific variables contained missing data, the proportion of missingness for each variable, and the particular methodology employed for the multiple imputation procedure (e.g., number of imputations, imputation model specifications). We recommend providing these technical details to enhance the transparency and reproducibility of the study.

11. Spelling and Grammatical Errors

Several spelling and typographical errors are present throughout the manuscript. We recommend comprehensive proofreading of the entire text, with specific attention to the following corrections:

In the Abstract, "Acut non-fulminantmyocarditis" should be corrected to "Acute non-fulminant myocarditis";

In the Abstract, "emergency departemnt" should be corrected to "emergency department";

In the Keywords section, "Acut non-fulminantmyocarditis" should be corrected to "Acute non-fulminant myocarditis";

In the Table 1 header, "IQR,interquartile rangen" should be corrected to "IQR, interquartile range".

Reviewer #4: Title: Clinical characteristics and emergency department-based warning indicators of

fulminant myocarditis in children

This is a clinically relevant retrospective study addressing early identification of fulminant myocarditis (FM) in children using variables available at emergency department (ED) presentation. The topic is important, particularly for resource-limited settings and frontline decision-making.

The manuscript is generally well-structured and includes appropriate statistical methods (LASSO + multivariable regression). However, several methodological and interpretive issues need clarification before the findings can be considered robust and generalizable.

Major concerns:

1. The study includes both patients who presented directly to the authors’ emergency department and those who were transferred from other hospitals to the ED prior to admission. This raises important concerns regarding the validity of the “ED-based” predictors. For transferred patients, the initial clinical presentation and laboratory values would have been obtained at the referring institution, not at the study ED. Consequently, the variables used in the analysis may reflect a later stage of disease progression, rather than the true initial ED presentation. This undermines the premise that the identified indicators are early warning signs available at first medical contact. Furthermore, management prior to transfer (e.g., fluids, vasoactive support, ventilation) may have significantly altered laboratory and clinical parameters.

Specify whether the analyzed variables represent first medical contact values, or values obtained after transfer.

2. the definition of FM is vague “On the basis of a diagnosis of acute myocarditis, patients who developed severe hemodynamic deterioration, cardiogenic shock, or life-threatening arrhythmias within a short period and required vasoactive agents or mechanical circulatory support were classified as FM”. The term “short period” is not defined, which introduces subjectivity and potential inconsistency in classification. In addition, life-threatening arrhythmias (ventricular tachycardia, high-grade AV block) can also occur in non-fulminant myocarditis, and are not specific to FM. The same goes for the need for intubation or circulatory/vasoactive support, which may also be observed in severe cases of non-fulminant myocarditis, particularly in tertiary care settings. As such, the current definition risks misclassification bias and may overlap significantly with markers of disease severity rather than representing a distinct clinical entity.

In addition, because several predictors identified in the model (e.g., lactate, AV block) are themselves markers of hemodynamic compromise, there is a risk of incorporation bias, where predictors and outcome definitions are not fully independent.

I suggest providing a clear and objective definition of “short period” (hours vs days from symptom onset or ED presentation), clarify whether FM classification was based on standardized criteria or institutional practice.

3. why was CMR not routinely performed?

4. Important clinical variables (e.g., blood pressure, LVEF, shock status) were excluded. While the authors justify this, these factors are critical determinants of severity and may confound associations. Either include these variables or clearly emphasize that the model reflects early risk markers rather than independent predictors.

5. While predictors are identified, the study does not provide Risk thresholds, Cut-off values, or a clinical scoring system. This limits translation into clinical practice. Please provide ROC analysis with optimal cutoffs if possible. Consider developing a simple clinical risk score.

Minor comments:

1. Language and grammar: Multiple typographical errors (e.g., “departemnt”, “Acut non-fulminantmyocarditis”).

2. Timing of data collection: Some variables were collected within 24 hours after admission, which may not reflect true ED presentation variables. Clarify timing and restrict to ED data if possible.

3. ECG findings: AV block is grouped together; consider stratifying by degree.

4. Troponin interpretation OR per unit increase is difficult to interpret clinically; consider transformation or clinically meaningful increments.

5. Figures: Ensure high resolution and improved clarity of LASSO plots.

6. Abbreviations: Standardize terminology (e.g., Lac vs lactate, cTnI formatting).

Reviewer #5: Early detection in practice remains challenging due to the nonspecific nature of early clinical manifestations, and the work does not clearly articulate the potential clinical impact of the findings: morbidity or mortality; apart from its prediction for fulminant cases.

Furthermore, the relative importance of the identified predictors is not well established, as no incremental values or comparisons among these factors are provided.

The applicability of the results is slightly overstated, and the conclusions would be strengthened by more cautious language—such as indicating that these factors "may help identify high-risk patients"—and by emphasizing the need for validation in prospective, multicenter studies.

**********

what does this mean?). If published, this will include your full peer review and any attached files.

If you choose “no”, your identity will remain anonymous but your review may still be made public.

**Do you want your identity to be public for this peer review?**  For information about this choice, including consent withdrawal, please see our Privacy Policy

Reviewer #1: No

Reviewer #2: No

Reviewer #3: No

Reviewer #4: **Yes:**  Rana Zareef

Reviewer #5: No

**********

[NOTE: If reviewer comments were submitted as an attachment file, they will be attached to this email and accessible via the submission site. Please log into your account, locate the manuscript record, and check for the action link "View Attachments". If this link does not appear, there are no attachment files.]

To ensure your figures meet our technical requirements, please review our figure guidelines: https://journals.plos.org/plosone/s/figures

You may also use PLOS’s free figure tool, NAAS, to help you prepare publication quality figures: https://journals.plos.org/plosone/s/figures#loc-tools-for-figure-preparation.

NAAS will assess whether your figures meet our technical requirements by comparing each figure against our figure specifications.

# Author comment: Author response to Decision Letter 1

**DOI:** 10.1371/journal.pone.0358335.r002

15 May 2026

Please see the attachment ”Response to Reviewers“.

Attachment Submitted filename: Response to Reviewers.docx

# Peer review: Decision Letter 1

**DOI:** 10.1371/journal.pone.0358335.r003

**Authors:** Hany Abo-Haded

20 Jul 2026

Dear Dr. Song,

Thank you for submitting your manuscript to PLOS ONE. After careful consideration, we feel that it has merit but does not fully meet PLOS ONE’s publication criteria as it currently stands. Therefore, we invite you to submit a revised version of the manuscript that addresses the points raised during the review process.

Please submit your revised manuscript by Sep 02 2026 11:59PM. If you will need more time than this to complete your revisions, please reply to this message or contact the journal office at plosone@plos.org. When you're ready to submit your revision, log on to https://www.editorialmanager.com/pone/ and select the 'Submissions Needing Revision' folder to locate your manuscript file.

- A letter that responds to each point raised by the academic editor and reviewer(s). You should upload this letter as a separate file labeled 'Response to Reviewers'.
- A marked-up copy of your manuscript that highlights changes made to the original version. You should upload this as a separate file labeled 'Revised Manuscript with Track Changes'.
- An unmarked version of your revised paper without tracked changes. You should upload this as a separate file labeled 'Manuscript'.

If you would like to make changes to your financial disclosure, please include your updated statement in your cover letter. Guidelines for resubmitting your figure files are available below the reviewer comments at the end of this letter.

If applicable, we recommend that you deposit your laboratory protocols in protocols.io to enhance the reproducibility of your results. Protocols.io assigns your protocol its own identifier (DOI) so that it can be cited independently in the future. For instructions see: https://journals.plos.org/plosone/s/submission-guidelines#loc-laboratory-protocols. Additionally, PLOS ONE offers an option for publishing peer-reviewed Lab Protocol articles, which describe protocols hosted on protocols.io. Read more information on sharing protocols at https://plos.org/protocols?utm_medium=editorial-email&utm_source=authorletters&utm_campaign=protocols.

As the corresponding author, your ORCID iD is verified in the submission system and will appear in the published article. PLOS supports the use of ORCID, and we encourage all coauthors to register for an ORCID iD and use it as well. Please encourage your coauthors to verify their ORCID iD within the submission system before final acceptance, as unverified ORCID iDs will not appear in the published article. *Only*  the individual author can complete the verification step; PLOS staff *cannot*  verify ORCID iDs on behalf of authors.

We look forward to receiving your revised manuscript.

Kind regards,

Hany Mahmoud Abo-Haded, MD

Academic Editor

PLOS One

**Journal Requirements:**

1. If the reviewer comments include a recommendation to cite specific previously published works, please review and evaluate these publications to determine whether they are relevant and should be cited. There is no requirement to cite these works unless the editor has indicated otherwise.

2. Please review your reference list to ensure that it is complete and correct. If you have cited papers that have been retracted, please include the rationale for doing so in the manuscript text, or remove these references and replace them with relevant current references. Any changes to the reference list should be mentioned in the rebuttal letter that accompanies your revised manuscript. If you need to cite a retracted article, indicate the article’s retracted status in the References list and also include a citation and full reference for the retraction notice.

[Note: HTML markup is below. Please do not edit.]

Reviewers' comments:

Reviewer's Responses to Questions

**Comments to the Author**

Reviewer #2: All comments have been addressed

**********

2. Is the manuscript technically sound, and do the data support the conclusions?

Reviewer #2: Yes

**********

3. Has the statistical analysis been performed appropriately and rigorously?

Reviewer #2: Yes

**********

4. Have the authors made all data underlying the findings in their manuscript fully available?

The PLOS Data policy

Reviewer #2: Yes

**********

5. Is the manuscript presented in an intelligible fashion and written in standard English?

Reviewer #2: Yes

**********

**Reviewer #2:**  The authors have substantially improved the revised manuscript and have addressed most of the major methodological concerns raised during the previous review round. The revised definition of fulminant myocarditis, clarification of first-medical-contact data acquisition, stratification of atrioventricular block, addition of ROC/AUC, calibration assessment, bootstrap internal validation, and supplementary analysis including LVEF have strengthened the manuscript. The study remains clinically relevant because the proposed warning indicators; gastrointestinal symptoms, elevated lactate, elevated cTnI, decreased sodium, and third-degree atrioventricular block are readily available during early emergency evaluation. The conclusions are now appropriately cautious and generally supported by the presented data. Should be corrected before acceptance:

1-Data availability statement inconsistency

The submission form indicates that some restrictions apply to data availability, whereas the manuscript states that all relevant data are included in the paper and Supporting Information files, including the anonymized minimal dataset as S1 Data.xlsx. These statements should be made fully consistent. If the dataset is fully available, the submission form should reflect this. If restrictions apply, the nature of the restrictions should be explicitly stated.

2- Residual language and typographical errors

The manuscript still contains several typographical and grammatical errors that should be corrected before publication. Examples include:

“abserved” should be “observed”

“Therefor” should be “Therefore”

“porgression” should be “progression”

“exernal validation” should be “external validation”

“discriminaton” should be “discrimination”

spacing errors such as “(1).Pediatric” and “e.g.,echocardiography”

inconsistent capitalization/formatting of cTnI/CTnI

duplicated wording in the Limitations section: “Future prospective multicenter studies are needed to validate the robustness and generalizability of these findings across Future prospective multicenter studies…”

the manuscript should undergo careful language editing.

3-Clarify terminology: warning indicators vs prediction model

The authors correctly state that this study identifies early warning indicators rather than developing a formal prediction model. This distinction should remain consistent throughout the manuscript. Terms such as “predictive model” or “model performance” should be used cautiously unless clearly referring only to the discriminatory performance of the indicator combination.

4- Interpretation of incorporation bias

The authors now acknowledge that lactate and third-degree AV block may partly reflect hemodynamic compromise and may overlap with the clinical definition of fulminant myocarditis. This limitation is appropriate and should remain clearly stated. The conclusions should continue to avoid implying causal or independently predictive effects.

5- Minor table and figure formatting

Tables and figures are generally informative, but final formatting should be checked for journal consistency, especially abbreviations, spacing, decimal formatting, and image resolution.

**********

what does this mean?). If published, this will include your full peer review and any attached files.

If you choose “no”, your identity will remain anonymous but your review may still be made public.

**Do you want your identity to be public for this peer review?**  For information about this choice, including consent withdrawal, please see our Privacy Policy

Reviewer #2: No

**********

[NOTE: If reviewer comments were submitted as an attachment file, they will be attached to this email and accessible via the submission site. Please log into your account, locate the manuscript record, and check for the action link "View Attachments". If this link does not appear, there are no attachment files.]

To ensure your figures meet our technical requirements, please review our figure guidelines: https://journals.plos.org/plosone/s/figures

You may also use PLOS’s free figure tool, NAAS, to help you prepare publication quality figures: https://journals.plos.org/plosone/s/figures#loc-tools-for-figure-preparation.

NAAS will assess whether your figures meet our technical requirements by comparing each figure against our figure specifications.

# Author comment: Author response to Decision Letter 2

**DOI:** 10.1371/journal.pone.0358335.r004

30 Jul 2026

Please see the attached "Response to Reviewer" document for our detailed point-by-point responses to all comments. All corresponding revisions have been incorporated into the revised manuscript and are highlighted in blue.

Attachment Submitted filename: Response_to_Reviewers_auresp_2.docx

# Peer review: Decision Letter 2

**DOI:** 10.1371/journal.pone.0358335.r005

**Authors:** Hany Abo-Haded

1 Sep 2026

Clinical characteristics and emergency department-based warning indicators of fulminant myocarditis in children

PONE-D-26-05939R2

Dear Dr. Song,

We’re pleased to inform you that your manuscript has been judged scientifically suitable for publication and will be formally accepted for publication once it meets all outstanding technical requirements.

Within one week, you’ll receive an e-mail detailing the required amendments. When these have been addressed, you’ll receive a formal acceptance letter and your manuscript will be scheduled for publication.

An invoice will be generated when your article is formally accepted. Please note, if your institution has a publishing partnership with PLOS and your article meets the relevant criteria, all or part of your publication costs will be covered. Please make sure your user information is up-to-date by logging into Editorial Manager at Editorial Manager® and clicking the ‘Update My Information' link at the top of the page. For questions related to billing, please contact billing support.

If your institution or institutions have a press office, please notify them about your upcoming paper to help maximize its impact. If they’ll be preparing press materials, please inform our press team as soon as possible -- no later than 48 hours after receiving the formal acceptance. Your manuscript will remain under strict press embargo until 2 pm Eastern Time on the date of publication. For more information, please contact onepress@plos.org.

Kind regards,

Hany Mahmoud Abo-Haded, MD

Academic Editor

PLOS One

Additional Editor Comments (optional):

Reviewers' comments:

Reviewer's Responses to Questions

**Comments to the Author**

Reviewer #2: All comments have been addressed

**********

2. Is the manuscript technically sound, and do the data support the conclusions?

Reviewer #2: Yes

**********

3. Has the statistical analysis been performed appropriately and rigorously?

Reviewer #2: Yes

**********

4. Have the authors made all data underlying the findings in their manuscript fully available?

The PLOS Data policy

Reviewer #2: Yes

**********

5. Is the manuscript presented in an intelligible fashion and written in standard English?

Reviewer #2: Yes

**********

Reviewer #2: The authors have adequately addressed my previous comments. The revised manuscript is substantially improved, and I have no remaining scientific concerns that would preclude publication.

One minor numerical correction may be addressed during final editorial processing: in Table 1, mortality is reported as 5 (9.8%), whereas 5/54 corresponds to 9.3%, consistent with the Results section.

**********

what does this mean?). If published, this will include your full peer review and any attached files.

If you choose “no”, your identity will remain anonymous but your review may still be made public.

**Do you want your identity to be public for this peer review?**  For information about this choice, including consent withdrawal, please see our Privacy Policy

Reviewer #2: No

**********

# Editor report: Acceptance letter

**DOI:** 10.1371/journal.pone.0358335.r006

**Authors:** Hany Abo-Haded

PONE-D-26-05939R2

PLOS One

Dear Dr. Song,

I'm pleased to inform you that your manuscript has been deemed suitable for publication in PLOS One. Congratulations! Your manuscript is now being handed over to our production team.

At this stage, our production department will prepare your paper for publication. This includes ensuring the following:

* All references, tables, and figures are properly cited

* All relevant supporting information is included in the manuscript submission,

* There are no issues that prevent the paper from being properly typeset

You will receive further instructions from the production team, including instructions on how to review your proof when it is ready. Please keep in mind that we are working through a large volume of accepted articles, so please give us a few days to review your paper and let you know the next and final steps.

Lastly, if your institution or institutions have a press office, please let them know about your upcoming paper now to help maximize its impact. If they'll be preparing press materials, please inform our press team within the next 48 hours. Your manuscript will remain under strict press embargo until 2 pm Eastern Time on the date of publication. For more information, please contact onepress@plos.org.

You will receive an invoice from PLOS for your publication fee after your manuscript has reached the completed accept phase. If you receive an email requesting payment before acceptance or for any other service, this may be a phishing scheme. Learn how to identify phishing emails and protect your accounts at https://explore.plos.org/phishing.

If we can help with anything else, please email us at customercare@plos.org.

Thank you for submitting your work to PLOS One and supporting open access.

Kind regards,

PLOS One Editorial Office Staff

on behalf of

Professor Hany Mahmoud Abo-Haded

Academic Editor

PLOS One
